# Occurrence and Risk Assessment of Tetracyclines and Their Transformation Products in Organic Amendments

**DOI:** 10.3390/antibiotics15090865

**Published:** 2026-09-04

**Authors:** Noelia García-Criado, Juan Luis Santos, Julia Martín, Irene Aparicio, Esteban Alonso

**Affiliations:** Departamento de Química Analítica, CATEPS, Escuela Politécnica Superior, Universidad de Sevilla, E-41092 Seville, Spain; ngarcia5@us.es (N.G.-C.); jlsantos@us.es (J.L.S.); jbueno@us.es (J.M.); iaparicio@us.es (I.A.)

**Keywords:** antibiotics, tetracyclines, transformation products, livestock manure, sewage sludge, environmental risk assessment

## Abstract

**Background/Objectives**: The application of organic soil amendments constitutes an important source of antibiotic residues in agricultural soils. However, studies have mainly focused on a few parent tetracyclines, whereas information on their transformation products (TPs) in organic amendments remains scarce. Therefore, this study assessed the occurrence and environmental risk of six tetracyclines and seven TPs in organic amendments. **Methods**: Processed livestock manures applied in the European Union (horse, poultry, and bovine manure), as well as fresh and treated sewage sludge, were analyzed using matrix solid-phase dispersion (MSPD) combined with online SPE-LC-MS/MS. Environmental risk was assessed using risk quotients (RQs) based on predicted environmental concentrations in soil (PEC_soil_) calculated from the maximum measured concentrations and predicted no-effect concentrations in soil (PNEC_soil_) obtained either directly from terrestrial ecotoxicity data or derived from aquatic ecotoxicity data using the equilibrium partitioning method and soil-water distribution coefficients. **Results**: Tetracyclines and their TPs were widely detected in both sample types, although concentrations varied according to matrix type and treatment. Overall, sludge showed higher detection frequencies and concentrations than manure. Doxycycline and tetracycline were the predominant parent compounds, reaching concentrations up to 2838 ng g^−1^ dry weight (dw) in manure and 2892 ng g^−1^ dw in sludge. Epimerized TPs were frequently detected and sometimes exceeded the concentrations of their parent compounds, especially epitetracycline and epioxytetracycline. Among the sludge types and treatment conditions investigated, anaerobically digested sludge showed the highest tetracycline concentrations, whereas the composted sludge sample presented the lowest concentrations. Individual RQs indicated insignificant to low ecotoxicological risk, whereas cumulative RQ (ΣRQ) values, used as conservative estimates of co-exposure to all evaluated tetracyclines and their TPs, fell within the medium-risk category for bovine manure and anaerobically digested sludge, with the composted sludge sample showing the lowest ΣRQ. **Conclusions**: These findings highlight the importance of including TPs in environmental monitoring and the differences in tetracycline occurrence and environmental risk to soil among the processed manure types and sludge treatment conditions investigated.

## 1. Introduction

Among the different antibiotic classes, tetracyclines are of particular environmental interest because of their extensive use in both human and veterinary medicine, as well as their physicochemical properties, which influence their environmental behavior. These compounds represent approximately 10–12% of the global antimicrobial market [1]. In human medicine, tetracyclines typically account for around 5–10% of total antibiotic consumption in most countries, whereas in veterinary medicine they represented the most widely used antimicrobial class worldwide in 2024, accounting for 29%, according to data reported by the World Organisation for Animal Health [2]. After administration, tetracyclines used in human medicine are usually discharged into the sewer system. In veterinary medicine, they can reach the sewer system or even be released into the soil. Tetracyclines such as tetracycline (TC), oxytetracycline (OTC), chlortetracycline (CTC), doxycycline (DOX), minocycline (MNC) and methacycline (MTC) are amphoteric compounds with affinity for organic matter and polyvalent cations, which promotes their sorption onto solid matrices such as sludge, manure, and soils [3]. Consequently, although tetracyclines may be detected in the aqueous phase, they tend to accumulate preferentially in solid environmental compartments, where they may persist and act as a source of contamination over time.

In addition to the parent compounds, tetracycline transformation products (TPs) are recognized as environmentally relevant contaminants [4]. These products are formed through processes such as epimerization, dehydration, isomerization, and biodegradation, giving rise to compounds including 4-epimers, iso-derivatives, and anhydro-derivatives [5,6]. These TPs may account for a substantial fraction of the total tetracycline burden in environmental matrices and, in some cases, may approach or exceed the concentrations of the parent compounds [6,7]. These transformation processes may also occur during manure storage and treatment and throughout wastewater treatment. Zhong et al. [6] described TP formation through abiotic hydrolysis and photolysis, as well as microbial degradation under aerobic and anaerobic conditions during wastewater treatment, while Wu et al. [7] reported tetracycline transformation during manure storage and thermophilic composting. In both studies, anhydrotetracycline (ATC), 4-epianhydrotetracycline (EATC), epitetracycline (ETC), epioxytetracycline (EOTC), 4-epichlortetracycline (ECTC), and isochlortetracycline (ICTC) were detected, with ETC, ECTC, and EOTC among the predominant TPs. Moreover, Schueler et al. [8] detected ETC, ATC, and ECTC during on-farm composting of dairy cattle manure and observed changes in their concentrations throughout composting. Some TPs also retain antibacterial activity and may contribute to environmental persistence through interconversion processes, particularly between tetracyclines and their 4-epimers [5].

Due to their widespread use, physicochemical properties and transformation processes, both tetracyclines and their TPs have been reported in a wide range of environmental compartments. Their occurrence and behavior depend on the environmental matrix. In wastewater treatment plants (WWTPs), these compounds have been detected throughout the treatment line, including influent, effluent, and sludge, reflecting inputs mainly associated with human use [6,9]. Particular attention has been given to livestock-derived matrices, where concentrations are generally higher due to the extensive use of tetracyclines in veterinary medicine and their subsequent excretion in urine and faeces [4]. As a result, tetracyclines and their TPs accumulate in manure, composted materials, and other organic amendments. The application of sludge and manure to agricultural soils represents a major pathway for the introduction of these contaminants into the terrestrial environment, where they may persist, transform, and potentially impact soil systems [10]. Among environmental matrices, sewage sludge and livestock manure are considered two of the major sources of tetracycline inputs into agricultural soils due to their widespread use as organic amendments. Current European legislation regulating the agricultural use of sewage sludge is primarily based on Council Directive 86/278/EEC [11], which mainly focuses on heavy metals and does not establish harmonized limit values for most organic micropollutants, including antibiotics and their TPs. Several Member States have adopted additional national requirements, including stricter limits for metals and, in some cases, limit values for selected organic contaminants such as PAHs, PCBs, PCDD/Fs, AOX, DEHP, LAS, and NP/NPE [12]. However, specific regulatory thresholds for antibiotics, including tetracyclines, in sewage sludge remain largely unavailable.

The environmental implications of tetracyclines and their TPs are related to their persistence and accumulation in environmental matrices, particularly in soils amended with manure and other organic residues. In addition, TPs, especially 4-epimers and anhydro-derivatives, may represent a relevant fraction of total residues. Some TPs retain antibacterial activity, while interconversion with their parent compounds may further contribute to their environmental persistence [4,13]. Tetracyclines may affect soil microbial functions. DOX has been reported to temporarily inhibit nitrogen transformation processes and alter soil microbial activity, suggesting potential effects on microbial community structure [14]. Tetracyclines may also affect terrestrial organisms. Phytotoxic effects on plant growth and root development have been reported [15], while CTC has been shown to induce reproductive, biochemical, and genotoxic effects in the soil invertebrate *Eisenia fetida* [16]. These responses may vary depending on the compound, species, and exposure level. Risk assessment studies indicate that including TPs can increase the estimated ecological risk relative to assessments based solely on parent compounds, particularly in terrestrial systems [4,17]. However, several environmental risk assessments (ERA) have focused exclusively on parent compounds and have frequently been restricted to aquatic compartments [18,19].

Despite the growing body of literature on tetracycline occurrence in environmental matrices, most studies have focused primarily on the parent compounds, and aquatic compartments, whereas TPs remain comparatively overlooked. In particular, information regarding the occurrence and environmental risk of tetracycline TPs in sludge and livestock manures remains limited, despite the relevance of these matrices as organic amendments and potential sources of soil contamination.

Therefore, a comprehensive assessment of tetracyclines in environmental matrices should include not only the parent antibiotics but also their main TPs. Such an approach is essential to improve our understanding of their occurrence, transformation pathways, persistence, and environmental relevance in both wastewater-related and livestock-derived systems. In this context, this study aimed to evaluate the occurrence and distribution of tetracyclines and their main TPs in livestock manure and sewage sludge, the two major sources of tetracycline inputs to agricultural soils, and to assess their potential environmental risk based on the measured concentrations.

The novelty of this study lies in the integrated assessment of parent tetracyclines and their TPs across different types of amendments and sludge treatment conditions investigated, alongside the associated risk assessment. This integrated approach revealed higher detection frequencies and concentrations in sludge than in manure, and generally insignificant to low ecotoxicological risks depending on amendment type and treatment conditions.

## 2. Results and Discussion

### 2.1. Occurrence of Tetracyclines in Livestock Manures

The concentrations of tetracycline antibiotics and their TPs were determined in processed livestock manure samples to assess their occurrence and potential input into manure-amended agricultural soils. The concentrations measured in the studied manures are summarized in Table 1. All parent compounds, except MTC and MNC, were detected in the studied manure samples. This observation is consistent with current patterns of veterinary antimicrobial use in the European Union, where OTC, CTC, TC and DOX are the tetracyclines most commonly applied to food-producing animals [20], while MNC and MTC are not authorised for veterinary use in food-producing animals and do not appear in the Union Product Database maintained by the European Medicines Agency [21]. Three of the six studied TPs were quantified, most of them at concentrations higher than those measured for their parent compounds. The TPs ATC, EATC and ECTC were not detected in any of the analyzed samples. Measured concentrations ranged from 4.1 (ETC) to 2838 ng g^−1^ (DOX), both concentrations measured in bovine manure. However, the frequencies of detection were low (25%: TC, ETC, CTC, ICTC; 42%: DOX; 67%: EOTC; 75%: OTC).

Considering only the parent compounds, DOX, TC, OTC and CTC were quantified in the manure from all studied animals, except CTC in the case of poultry manure. The highest concentrations were measured for DOX (mean concentration 1457 ng g^−1^ dw), which were markedly higher than those measured for TC (54.5 ng g^−1^ dw), OTC (40.9 ng g^−1^ dw), and CTC (16.4 ng g^−1^ dry weight (dw)).

The concentrations measured in the present study fell within the broad range of tetracycline concentrations previously reported in processed manure-derived fertilizers and composts. Zhang et al. [22] reported maximum concentrations of 3700 and 3685 ng g^−1^ dw for TC and DOX, respectively, in refined commercial compost. In mature cattle and poultry manure composts, Xie et al. [23] reported concentrations of up to 32.7, 7.2, 57.6, and 30.1 ng g^−1^ dw for TC, OTC, CTC, and DOX, respectively. Zhou et al. [24] reported maximum concentrations of 1922, 81.8, 773.5, and 85.4 ng g^−1^ dw, respectively, in fertilizers derived from cow, swine, and chicken manure. Hong et al. [25] also reported tetracyclines in fertilizers derived from chicken, swine, sheep/goat, and cattle manure, with OTC reaching 110,000 ng g^−1^ dw. More recently, Wu et al. [7] reported maximum concentrations of 11,451, 55,007, 30,305, and 4348 ng g^−1^ dw for DOX, OTC, CTC, and TC, respectively, in manure-derived organic fertilizers. Among the TPs, ECTC and EOTC reached maximum concentrations of 31,310 and 12,772 ng g^−1^ dw, respectively.

Although processed manure-derived fertilizers have been particularly investigated in China, comparable data from Europe remain more limited. In Poland, Patyra et al. [26] detected antibiotics in 9 of 24 solid commercial organic fertilizers produced from cattle, horse, or sheep manure. Antibiotic concentrations ranged from 47.0 to 757.9 ng g^−1^, with OTC and its epimer among the most frequently detected compounds. By comparison, substantially higher tetracycline concentrations have been reported in untreated or fresh livestock manure in some studies. For instance, Conde-Cid et al. [3] reported maximum DOX concentrations of 106,000, 14,000, and 300 ng g^−1^ dw in pig, poultry, and cattle manure from Spain, respectively.

Information on TPs remains comparatively limited, as many previous occurrence studies have focused primarily on parent tetracyclines [3,22,23,24].

Differences in manure processing may contribute to the wide variability in tetracycline concentrations among studies. Wu et al. [7] observed average reductions of 82% and 90% in total parent tetracyclines and TPs, respectively, following thermophilic composting, whereas manure storage did not result in a significant decrease. Similarly, Xie et al. [23] reported significantly lower total tetracycline concentrations in composts than in the corresponding raw manures.

The wide variability in tetracycline concentrations among studies may reflect differences in antibiotic usage patterns, animal species, routes of administration, manure collection time, dosage, as well as their physicochemical and pharmacokinetic properties [27]. For example, in this work, the highest DOX concentrations were measured in bovine and equine manures (Table 1). However, DOX is not among the most widely used tetracycline antibiotics in veterinary medicine. Other compounds such as OTC or TC are more widely used globally [1,20]. However, its presence in bovine manure, where it is less commonly used, could be associated with sporadic therapeutic applications and its high excretion rate in unchanged form [20]. Indeed, up to 90% of the administered dose may be recovered as the parent compound in urine and feces [1,4]. Its occurrence may also reflect a lower removal rate during manure treatment than that observed for other tetracyclines [28,29]. Although antibiotic use in cattle is generally lower than in poultry because of stricter control of veterinary drug residues in milk production [30], elevated DOX concentrations have also been reported in cattle manure [3].

In contrast, in the case of equine medicine, DOX is widely used, particularly for intracellular bacterial infections [31]. This could explain the high concentrations measured in these samples. However, its occurrence in bovine manure, where it is less commonly prescribed, could also be related to its persistence in manure matrices. Previous studies have reported comparatively low removal efficiencies for DOX during manure composting and suggested that DOX metabolites excreted by animals may be strongly associated with the manure matrix, reducing their susceptibility to degradation during manure treatment processes [29].

Considering the studied TPs, only the epimers were detected, while the anhydro forms showed concentrations below the detection limits in all the samples analyzed. Overall, the combined concentrations of TPs frequently exceeded those of their corresponding parent compounds (Figure S1). Based on mean concentrations in the manure types where both compounds were detected, EOTC accounted for 90–96% of the OTC-EOTC fraction (Figure S1a), whereas ICTC represented 72–73% of the CTC-ICTC fraction (Figure S1b). This pattern may reflect multiple transformation processes occurring throughout the manure production and processing chain, as reported previously [32]. Previous studies have also reported lower tetracycline concentrations in stored than in fresh manures, supporting the occurrence of degradation and transformation processes during manure storage [3]. Tetracyclines are amphoteric molecules with multiple ionizable functional groups, and their speciation depends strongly on matrix pH [3]. Under typical manure storage conditions, which generally range from near-neutral to slightly alkaline pH [24], epimerization processes may occur [5,6], which could explain the higher concentrations measured in this work. Therefore, the predominance of EOTC over OTC in some samples could be related to storage-induced transformation [6]. In addition to transformation processes, previous studies have highlighted the strong affinity of tetracyclines for organic matter and polyvalent cations (e.g., Ca^2+^ and Mg^2+^), which are abundant in livestock wastes [4]. The formation of chelation complexes and associations with organic constituents may reduce their mobility and favor their retention in the solid fraction of manure [4]. This behavior is consistent with the known sorption characteristics of tetracyclines in organic-rich matrices [4].

### 2.2. Occurrence of Tetracyclines and TPs in Sewage Sludge

**Figure 1:**
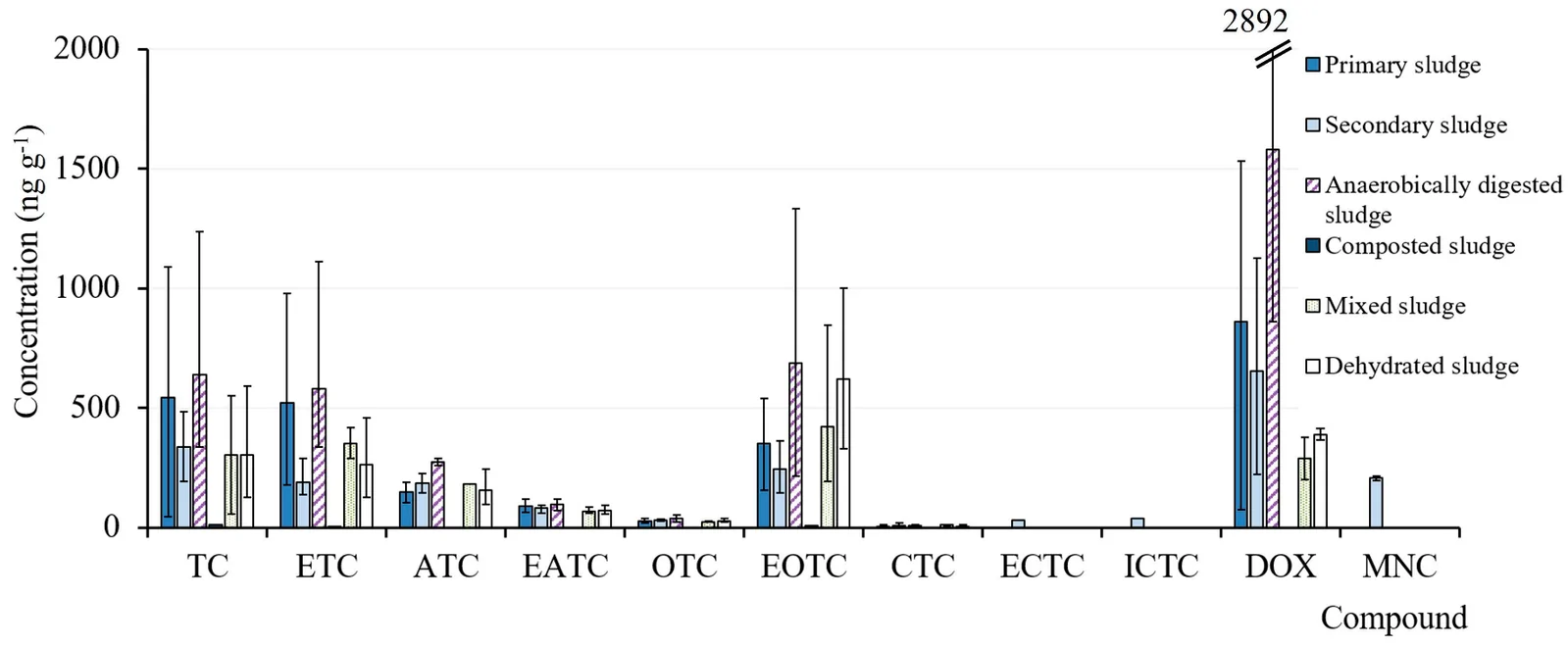
Concentrations of tetracyclines and their transformation products in fresh and treated sewage sludge samples.

The concentrations of tetracyclines and their TPs were determined in fresh sludge (primary, secondary and mixed sludge), and in the treated sludge most usually applied to the soil, including dehydrated, anaerobically digested, and composted sludge, to assess their occurrence prior to land application. Measured concentrations are summarized in Figure 1. Concentration ranges, frequency of detection and mean concentrations are shown in Table S1 (Supplementary Materials). All target compounds, except MTC, were detected in most of the studied sludges, with differences between fresh and treated materials. Their frequency of detection and concentrations were higher than those measured in manure samples. Only DOX showed, in one sample of bovine manure, a concentration higher than those measured in sludge samples. This can be explained considering the use of these antibiotics in veterinary and human medicine. While processed manures reflect veterinary administration followed by transformation driven by storage and processing, sewage sludge integrates diffuse inputs from domestic, hospital and industrial sources and undergoes sequential biological treatments [14,15].

Overall, lower mean concentrations were observed in dehydrated and composted sludge than in fresh sludge in this sampling campaign. In composted sludge, parent compounds were detected at lower concentrations, while some TPs remained detectable, albeit at low levels.

These findings show differences in the distribution and concentrations of tetracyclines and their TPs among the sludge types investigated, in agreement with previous observations reported by Mejías et al. [33]. Considering parent compounds, the highest concentrations were measured in the case of DOX and TC. In fresh sludge, their concentrations ranged from 74.2 to 1534 ng g^−1^ dw and from 44.2 to 1091 ng g^−1^ dw, respectively. The remaining parent compounds were measured at concentrations up to 34.2 ng g^−1^ dw in these samples. These concentrations were within the range previously reported in sewage sludge in several European countries. Salvia et al. [34] reported TC concentrations ranging from 31 to 135 ng g^−1^ dw in sludge samples, whereas Verlicchi and Zambello [35] reported DOX and TC concentrations above 560 ng g^−1^ dw in conditioned sludge. The occurrence of these compounds in sewage sludge may be related to the prescription of tetracyclines in human medicine [36]. Indeed, DOX is among the most prescribed tetracyclines in the European Union, mainly due to its higher oral bioavailability and reduced food interactions compared to other tetracyclines [20], followed by TC and OTC, whereas the use of CTC is less common [37].

Epimers were the most frequently detected TPs and at the highest concentrations. EOTC (up to 845 ng g^−1^ dw) showed concentrations higher than its parent compound and accounted for 74–90% of the combined OTC-EOTC fraction (Figure S2a), while the concentrations of ETC were similar to those measured for TC (up to 979 ng g^−1^ dw). Moreover, ICTC accounted for 50–81% of the CTC-related compounds in the sludge samples where both compounds were detected (Figure S2b). ATC and EATC were measured at concentrations ranging from 106 to 227 ng g^−1^ dw (in 42% of the fresh sludge samples analyzed) and from 59.0 to 119 ng g^−1^ dw (in 83% of the samples), respectively. Regarding the occurrence of target compounds across the sludge treatment processes investigated, the concentrations of most of the studied compounds (TC, ETC and EOTC) showed a similar distribution pattern, as illustrated in Figure S2. In anaerobic digestion and composting, the concentrations measured in primary sludge were higher than those measured in secondary sludge and both were lower than those measured after anaerobic digestion. Finally, the lowest concentrations of the studied compounds were observed in the composted sludge sample. More recently, Zhong et al. [6] investigated both parent tetracyclines and their TPs in three municipal WWTPs in Guangzhou, China, reporting concentrations of up to 261 ng g^−1^ for TC and 1190 ng g^−1^ dw for OTC in sewage sludge. Despite these reports, information on tetracycline TPs in sewage sludge remains limited, as previous occurrence studies have primarily focused on parent tetracyclines.

The concentrations measured in mixed and dehydrated sludges were similar for all studied compounds. Previous studies have reported degradation of these compounds under aerobic conditions [4,35,36], whereas lower degradation has been observed during anaerobic treatment [17,38]. Moreover, the lower concentrations measured in secondary than in primary sludge could be influenced by differences in their adsorption onto secondary sludge and by the aerobic conditions applied during the activated sludge process. The higher concentrations measured in anaerobically digested and dehydrated sludge samples could be explained by the persistence of these compounds during anaerobic treatment and by sludge weight loss during treatment and dehydration [35,38]. An exception to this behaviour was observed for ATC, OTC, and CTC, whose concentrations were higher in secondary than in primary sludge samples. This pattern could be attributed to differences in sorption behaviour between sludge matrices [4].

### 2.3. Environmental Risk Assessment

The analysis of treated products intended for land application, specifically manure and treated sewage sludge, showed that both TPs and parent compounds persist after these processes. Parent compounds were detected at high concentrations, with manure samples reaching up to 2838 ng g^−1^ dw, although with a low detection frequency (Table 1). Compounds such as TC and DOX were found at elevated concentrations in anaerobically digested sludge (up to 1238 and 2892 ng g^−1^ dw, respectively) and dehydrated sludge (up to 590 and 365 ng g^−1^ dw, respectively). Furthermore, some TPs that retain antimicrobial activity comparable to, or even exceeding, that of the parent compounds were also measured at high concentrations. ETC reached concentrations of up to 1113 and 459 ng g^−1^ dw in anaerobically digested and dehydrated sludge, respectively, while other TC TPs, such as ATC and EATC, showed concentrations of up to 291 and 119 ng g^−1^ dw in these samples. Additionally, EOTC showed concentrations of up to 1334 and 1002 ng g^−1^ dw in anaerobically digested and dehydrated sludge, respectively. These concentrations suggest that the application of manure and sludge to soil could result in a potential ecotoxicological risk, not only due to the presence of antibiotics in these amendments, but also due to their TPs. Consequently, ecotoxicological risks may be underestimated when considering only the parent compounds. Ecotoxicological data reported in the literature for the studied compounds are summarized in Table S2 (Supplementary Materials). Table 2 presents the selected ecotoxicological values reported in the recent literature for each target compound, together with the applied assessment factors, soil-water partition coefficients (*K*_d_), and the resulting PNEC_water_, equilibrium partitioning (EqP)-derived PNEC_soil_ and PNEC_soil_ calculated from terrestrial toxicity data. Terrestrial ecotoxicological data were only available for parent compounds. The EqP-derived PNEC_soil_ values were calculated using the selected ecotoxicological endpoints and ranged from 31 to 2528 ng g^−1^ for most target compounds, whereas EOTC and ICTC showed the lowest (31 ng g^−1^) and highest (25,700 ng g^−1^) values, respectively. For both compounds, the PNEC values were derived from the only experimental ecotoxicological endpoint available in the literature, based on sewage sludge bacteria. This highlights the scarcity of ecotoxicological data currently available for these TPs and the uncertainty associated with their risk assessment. For parent compounds, the PNEC_soil_ values calculated from terrestrial ecotoxicological endpoints for plants, earthworms and enchytraeids ranged from 96.1 to 6000 ng g^−1^. For all parent compounds, the PNEC_soil_ values based on terrestrial toxicity data were higher than the corresponding EqP-derived PNEC_soil_ values. The only exception was DOX, for which the terrestrial PNEC_soil_ value was lower, resulting in a more conservative risk assessment. Overall, the calculated PNEC_soil_ values provide a preliminary indication of the ecotoxicological hazard posed by tetracycline TPs.

For example, PNEC_soil_ values of 2484, 1758 and 2528 ng g^−1^ were determined for ETC, ATC and EATC, respectively, all of which were higher than that of their parent compound TC (56 ng g^−1^), suggesting a lower toxicity based on the currently available ecotoxicological data. However, experimentally derived terrestrial PNEC_soil_ values for most tetracycline TPs remain scarce, and current risk assessments frequently rely on equilibrium partitioning (EqP) approaches or QSAR-based estimations, introducing an additional source of uncertainty [7].

Table 3 shows the risk quotients (RQ) calculated for the different matrices using the most sensitive PNEC_soil_. The ERA in livestock manures revealed differences among the evaluated tetracyclines and matrices (Table 3). Overall, DOX showed the highest RQ values, particularly in bovine (RQ = 8 × 10^−2^) and horse (RQ = 6 × 10^−2^) manures, followed by poultry manure (RQ = 1.1 × 10^−2^). These findings are consistent with previous reports identifying DOX as one of the dominant tetracyclines in livestock manure and manure-amended soils due to its extensive veterinary use and environmental persistence [14,48]. TC also contributed to the overall risk profile, especially in poultry manure (RQ = 5 × 10^−3^), whereas OTC, CTC, and the remaining compounds exhibited lower RQ values. Among the TPs, EOTC was the main contributor to the overall risk profile, exhibiting low-risk levels in all manure types (RQ values ranged from 5 × 10^−2^ to 9 × 10^−2^), with the highest value observed in bovine manure. In contrast, ETC showed a substantially lower RQ value (2 × 10^−4^), corresponding to insignificant risk levels. According to the commonly accepted risk classification criteria, all evaluated compounds presented insignificant to low environmental risks (RQ < 0.1). Under this conservative screening approach of all tetracycline residues, ΣRQ values corresponded to low-risk categories for horse and poultry manure, whereas bovine manure fell within the medium-risk category. Nevertheless, the inclusion of TPs in ERA remains important because epimerized and anhydro derivatives are frequently co-excreted with their parent compounds and may contribute to the overall environmental burden despite often being overlooked in conventional monitoring studies [10,18]. Similar trends have been reported in previous studies, where bovine and swine manures exhibited higher tetracycline loads and risk indices than poultry manure [24,49]. Importantly, the RQ values obtained here were based on classical ecotoxicological endpoints and therefore refer to ecotoxicological risk.

The ERA of treated sludge samples indicated generally low risk (Table 3). Among the evaluated matrices, anaerobically digested sludge exhibited the highest RQ values, corresponding to low-risk levels, particularly for EOTC (RQ = 6 × 10^−2^), DOX (RQ = 4 × 10^−2^), and TC (RQ = 3 × 10^−2^). This was followed by dehydrated sludge, for which the highest RQ was observed for EOTC (RQ = 5 × 10^−2^), followed by TC (RQ = 2 × 10^−2^). The remaining parent compounds and TPs showed substantially lower RQ values, below 0.01, indicating insignificant environmental risks. In contrast, composted sludge presented the lowest ecotoxicological risk, with a maximum RQ value of 4 × 10^−3^ for EOTC and values below 3 × 10^−4^ for TC and its detected TP, ETC, all corresponding to insignificant risk levels. Among the sewage sludge samples and treatment conditions investigated, composted sludge showed the lowest estimated environmental risk associated with land application. The ΣRQ values corresponded to a medium-risk category for anaerobically digested sludge, low for dehydrated sludge, and insignificant for composted sludge.

This trend aligns with observations by Zhong et al. [6], who identified sludge as the compartment of ecological relevance in municipal WWTPs and demonstrated that integrating TPs into risk assessments considerably increases the risk profile. In their study, TPs accounted for 70–83% of the total tetracycline-related mass in sludge, with certain epimerized forms exceeding the concentrations of their parent compounds. The persistence and ecotoxicological relevance of these TPs have been linked to their poor biodegradability and, in specific instances, their retained antimicrobial potency [6,10,45].

Ghirardini et al. [18] reviewed the occurrence of antibiotics in cattle, swine, poultry and sheep manures and subsequently performed an ERA for a swine slurry-amended soil scenario. Using a PEC_soil_/PNEC_soil_ approach, the authors estimated PEC_soil_ values ranging from 64 to 2136 ng g^−1^ dw for TC, DOX, OTC and CTC under the highest manure application scenario and identified DOX and CTC as the compounds posing the highest ecotoxicological risk. However, the assessment was restricted to parent compounds and did not consider TPs, highlighting the limited information currently available regarding the environmental relevance of tetracycline TPs in livestock manures and manure-amended soils.

The limited availability of terrestrial ecotoxicological data for tetracycline TPs remains one of the main sources of uncertainty in their ERA. Therefore, future studies addressing the toxicity of epimers and anhydro-derivatives in soil organisms would improve the reliability and environmental relevance of risk assessments for manure- and sludge-amended soils.

An important source of uncertainty arises from applying parent-compound *K*_d_ values to the corresponding TPs, particularly EOTC and ICTC, because the actual sorption behavior of the TPs may differ. Although the lowest *K*_d_ values within the selected ranges were used to obtain conservative RQ estimates, experimental determination of compound-specific *K*_d_ values for individual TPs would allow a more accurate assessment of their environmental risk.

## 3. Materials and Methods

### 3.1. Chemicals and Reagents

High-purity analytical standards (>95%) of TC, OTC, CTC, DOX, MNC, and EATC were obtained from Sigma-Aldrich (Steinheim, Germany), whereas ETC, ECTC, and EOTC were purchased from Acros Organics (Geel, Belgium). Tetracycline TPs ATC, ICTC, MTC, and DMC were supplied by Toronto Research Chemicals (Toronto, ON, Canada). The chemical structures and relevant physicochemical properties of all target analytes are summarized in Table S3. LC–MS-grade ammonium fluoride was acquired from Honeywell (Seelze, Germany). LC–MS-grade methanol and ultrapure water were purchased from Biosolve BV (Valkenswaard, The Netherlands), while analytical-grade formic acid was obtained from Romil (Cambridge, UK). Ammonium formate, silica, and Florisil^®^ were supplied by Sigma-Aldrich (Steinheim, Germany), and primary–secondary amine (PSA) sorbent was provided by Scharlab (Barcelona, Spain). Ammonia solution (30% NH_3_) and reagents used for the preparation of the McIlvaine buffer-disodium hydrogen phosphate dihydrate, citric acid monohydrate, and disodium EDTA dihydrate, all analytical grade, were purchased from Panreac (Barcelona, Spain). Na_2_EDTA-McIlvaine buffer (pH 4.0) was prepared as follows: 13.73 g of Na_2_HPO_4_⋅2H_2_O was added to a 500 mL glass beaker and dissolved with pure water; then, 7.44 g of Na_2_EDTA and 12.92 g of citric acid monohydrate were added to the beaker, dissolved and diluted with pure water to 1 L in a volumetric flask; finally, the pH of the final solution was verified to ensure it was 4.0. Solid-phase extraction materials included empty 6-mL polypropylene SPE cartridges and 20-µm polyethylene frits from Supelco (Darmstadt, Germany). Regenerated-cellulose Captiva Premium syringe filters (4 mm, 0.2 µm) were supplied by Agilent Technologies (Santa Clara, CA, USA). Online SPE cartridge was obtained from Agilent Technologies (PLRP-S, 12.5 mm × 2.1 mm i.d., 15–20 µm). Individual stock solutions were prepared in MeOH at 1000 µg mL^−1^. Working solutions were obtained by appropriate dilution of the stock solutions with MeOH. All standard solutions were stored in amber glass vials at −18 °C until use.

### 3.2. Sampling

A total of eleven commercial manure samples were obtained from distributors in Spain, Germany, and Italy, representing three livestock species: cattle, poultry, and horses. All of them correspond to processed commercial manure products obtained from different distributors and were marketed in pelletized form. For some products, the manufacturers reported additional treatments such as drying, fermentation, stabilization, or controlled composting processes prior to commercialization.

Twenty-one sludge samples were collected from eight wastewater treatment plants (WWTPs) and one composting plant, distributed across the Andalusia region (southern Spain). Samples of fresh sludge (primary, secondary and mixed) and treated sludge (dehydrated, digested and composted) were collected.

Five samples each of primary and secondary sludge were collected from four anaerobic treatment plants and an aerobic treatment plant, whereas three mixed sludge samples were collected from dehydration treatment plants. Sludge treatments included dehydration of fresh sludge (in three dehydration treatment plants), anaerobic digestion (in four anaerobic treatment plants), and composting (in a composting plant). A total of 2,308,300 equivalent-inhabitants and an influent wastewater flow of 1,285,600 m^3^ day^−1^ were covered. A sampling campaign was carried out in April 2025. Samples were collected in duplicate at each sampling point, with the volumes or masses indicated below corresponding to each replicate. Two litres of each primary and secondary sludge, and 2 kg each of anaerobically digested and dehydrated sludge were collected from each of the four anaerobic treatment plants. Two litres of mixed and 1 kg of dehydrated sludge were collected from dehydration treatment plants and 2 kg of composted sludge from the composting plant. All samples, except composted sludge, were collected from sampling points located along the sludge treatment processes. Composted sludge was obtained from a sludge storage unit as a composite mixture of aliquots collected from various points within the unit.

Upon reception, all solid samples were freeze-dried using a Cryodos-50 lyophilizer (Telstar, Terrassa, Spain), homogenized using a mortar, finely ground, and sieved to a particle size < 1 mm. Processed samples were stored at −20 °C in amber glass containers until analysis.

### 3.3. Analysis and Quality Control

The extraction of the target compounds was performed by matrix solid-phase dispersion (MSPD). Online solid-phase extraction (online SPE) was applied as clean-up. Determination was carried out by liquid chromatography–tandem mass spectrometry (LC–MS/MS), as previously described by García-Criado et al. [50]. Further methodological details can be found in the Appendix A (Section Sample Treatment and Analysis) and in García-Criado et al. [50]. LC-MS/MS parameters applied to the MS determination can be found in Appendix A (Table A1).

Quantification was performed using a matrix-matched calibration curve by fortifying blank matrices at twelve concentration levels in the range of 0.5–2000 ng g^−1^ dw, with the validated linear range depending on the target compound [50]. Method performance was evaluated in terms of recovery, repeatability, linearity, and limits of detection and quantification. More details about the method performance can be found in García-Criado et al. [50]. A summary of the validation parameters is provided in Table S4 (Supplementary Materials). To ensure quality control, procedural blanks, matrix-fortified samples, and calibration standards were processed together with the samples for each analytical sequence. For each sequence, analytical parameters were estimated and compared with those obtained during the validation of the method. The sequence was considered acceptable when the analytical parameters did not differ by more than 20% from the validation values.

### 3.4. Environmental Risk Assessment

The ecotoxicological risk in soils amended with composted manures and treated sludges was assessed using risk quotient (RQ) values, according to the EMA, following the Guideline on the Environmental Risk Assessment of medicinal products for human use [51]. RQs were calculated as the ratio between the predicted environmental concentration of the target compounds in soil (PEC_soil_) and their corresponding predicted no-effect concentration (PNEC_soil_), following the quantitative approach described in the European Technical Guidance Document on Risk Assessment of the European Commission [52]. The PEC_soil_ values were calculated using a primary exposure model that assumes a single annual application of the amendment and complete mixing within the topsoil layer (0–20 cm) [52], rather than measured soil concentrations, to ensure comparability across matrices [53] using Equation (1):

PEC_soil_ = (C_amendment_ × APPL_amendment_)/(DEPTH_soil_ × RHO_soil_) (1)

where C_amendment_ (ng g^−1^ dw) is the measured concentration of the target compound in the applied amendment, APPL_amendment_ is the annual application rate (kg m^2^ yr^−1^) assumed to be 0.5 kg m^2^ yr^−1^ [52] for sludge and 0.9 kg m^2^ yr^−1^ [49] for manure, DEPTH_soil_ is the mixing depth (0.20 m), and RHO_soil_ is the bulk density of agricultural soil (1700 kg m^−3^) [52].

PNEC_soil_ values were derived using two complementary approaches depending on the availability of ecotoxicological data. When toxicity data for terrestrial soil organisms were available, PNEC_soil_ values were calculated as follows:

PNEC_soil_ = ecoTox_soil_/AF (2)

where ecoTox_soil_ represents the selected toxicity endpoint for terrestrial organisms and AF is the corresponding assessment factor.

Toxicity data from aquatic organisms were used to estimate PNEC_soil_ using the EqP approach [54]. First, PNEC_water_ was calculated according to Equation (3):

PNEC_water_ = ecoTox_water_/AF (3)

Subsequently, PNECsoil was estimated using Equation (4):

PNEC_soil_ = PNEC_water_ × *K*_d_ (4)

where ecoTox_water_ represents the selected aquatic toxicity endpoint and *K*_d_ is the soil-water partitioning coefficient (L kg^−1^).

Ecotoxicological endpoints (ecoTox) were selected according to the hierarchy recommended in the EMA guideline [51], prioritizing chronic (EC_10_ or NOEC) over acute toxicity data (EC_50_ or LC_50_) and EC_10_ over NOEC. When multiple values of the same endpoint type were available, the most sensitive value was selected. AF values of 10 were applied when chronic toxicity data (EC_10_ and NOEC) from three trophic levels were available, whereas AF values of 50 and 100 were applied when chronic data were available for two or one trophic levels, respectively. An AF of 1000 was applied when only acute toxicity data were available [52].

The ecotoxicological data and *K*_d_ values from the literature are compiled in Table 2. Risk levels were classified as insignificant (RQ < 0.01), low (0.01 ≤ RQ < 0.1), medium (0.1 ≤ RQ < 1), and high (RQ ≥ 1) [54]. For each amendment type, a cumulative risk quotient (ΣRQ) was calculated as the sum of the individual RQs for all evaluated tetracyclines and their TPs. The ΣRQ was used as a conservative screening measure for potential co-exposure to the evaluated compounds within each amendment type rather than as evidence of additive mixture toxicity, because the individual RQs were derived from different ecotoxicological endpoints and interactions among compounds were not considered. Additional details on the risk assessment procedure are provided in Appendix B Section Selection of Ecotoxicological Endpoints and Derivation of PNEC Values.

## 4. Conclusions

This study investigated the occurrence, distribution, and environmental risk to soil of tetracyclines and their main TPs in processed livestock manure and sewage sludge, including fresh and treated sludge subjected to dehydration, anaerobic digestion, and composting. The scope of the study was limited to processed commercial manure products and a single sewage sludge sampling campaign, with composted sludge represented by one facility. Consequently, temporal or seasonal variability was not evaluated in the present study. Tetracyclines and their TPs were widely detected in both studied matrices, although sewage sludge generally showed higher detection frequencies and concentrations than livestock manure. DOX and TC were the predominant parent compounds, while epimerized TPs frequently occurred at concentrations comparable to, or even higher than, those of their corresponding parent compounds. Differences in the distribution and concentrations of tetracyclines and their TPs were observed among the sludge types and treatment conditions investigated. Among the samples analyzed, anaerobically digested sludge exhibited the highest concentrations and RQ values, whereas the composted sludge sample showed the lowest concentrations and ecotoxicological risks following land application. Individual RQ values for manure and sludge samples corresponded to insignificant or low ecotoxicological risk levels. These RQs were derived from ecotoxicological endpoints; therefore, the low values observed do not provide information on the potential for antimicrobial resistance selection. Nevertheless, several TPs remained detectable in treated sludge samples. These findings highlight the environmental importance of considering tetracycline TPs in environmental monitoring and risk assessment studies, given their frequent occurrence and persistence in the sludge samples analyzed. Furthermore, these findings support regular monitoring of tetracyclines and their TPs in agricultural amendments. Further ecotoxicological studies and compound-specific (*K*_d_) measurements, particularly for tetracycline TPs, are needed to reduce current uncertainties in terrestrial risk assessment and support future regulatory frameworks.

## Figures and Tables

**Table 1:** Concentration ranges, occurrence (frequency of detection) and mean concentrations of antibiotics in studied amendments.

Compound	Amendment Concentration								
	Horse (n = 3)			Poultry (n = 4)			Bovine (n = 4)		
	Mean ^1^ (ng g^−1^)	Range (ng g^−1^)	Occ. (%)	Mean ^1^ (ng g^−1^)	Range (ng g^−1^)	Occ. (%)	Mean ^1^ (ng g^−1^)	Range (ng g^−1^)	Occ. (%)
TC	27.3	<MDL-27.3	33	107	<MDL-107	25	29.4	<MDL-29.4	25
*ETC*	38.9	<MDL-38.9	33	95.4	<MDL-95.4	25	4.1	<MDL-4.1	25
*ATC*	-	-	0	-	-	0	-	-	0
*EATC*	-	-	0	-	-	0	-	-	0
OTC	67.1	<MDL-113	67	18.7	<MDL-21.1	50	37	16.8–83.4	100
*EOTC*	617	<MDL-617	33	466	<MDL-713	75	597	<MDL-1069	75
CTC	15.5	<MDL-15.5	33	-	-	0	17.2	<MDL-17.2	25
*ECTC*	-	-	0	-	-	0	-	-	0
*ICTC*	42.4	<MDL-42.4	33	-	-	0	44.5	<MDL-44.5	25
DOX	1131	4.1–2161	100	402	<MDL-402	25	2838	<MDL-2838	25
MTC	-	-	0	-	-	0	-	-	0
MNC	-	-	0	-	-	0	-	-	0

^1^ Mean concentrations of quantified samples only; TPs are marked in italics. Concentrations are reported as ng g^−1^ dry weight (dw). Occ.: Occurrence; -: <MDL.

**Table 2:** Lowest ecotoxicological data, predicted no-effect concentration (PNEC) in water and soil, and soil-water distribution coefficient (*K*_d_) of target compounds.

Compound	Species	Endpoint	Effect	Ref.	AF	PNEC_aquatic_ (mg L^−1^)	PNEC_soil_ (ng g^−1^)	*K*_d_ ^a^ (L kg^−1^)
TC	*Daucus carota*	EC_10_	0.0019 mg L^−1^	[39]	10	0.00019	56–523	295–2754
	*Cucumis sativus*	EC_10_ (RL)	300 mg kg^−1^	[40]	100	-	3000 ^b^	-
*ETC*	*Chlorella vulgaris*	EC_50_ (IR)	8.42 mg L^−1^	[41]	1000	0.00842	2484–23,189	295–2754
*ATC*	*Chlorella vulgaris*	EC_50_ (IR)	5.96 mg L^−1^	[41]	1000	0.00596	1758–16,414	295–2754
*EATC*	*Danio rerio embryos*	EC_50_ (M)	8.57 mg L^−1^	[42]	1000	0.00857	2528–23,602	295–2754
OTC	*Ceriodaphnia silvestrii*	EC_10_	0.04 mg L^−1^	[43]	10	0.004	460–9596	115–2399
	*Enchytraeus crypticus*	EC_10_	134 mg kg^−1^	[44]	100		1340 ^b^	-
*EOTC*	Aerobic activated sludge bacterial community	EC_50_	0.27 mg L^−1^	[45]	1000	0.00027 ^c^	31–648	115–2399
CTC	*Daucus carota*	EC_10_ (RL)	0.0031 mg L^−1^	[39]	50	0.000062	44–230	708–3715
	*Cucumis sativus*	EC_10_ (RL)	300 mg kg^−1^	[40]	50		6000 ^b^	-
*ICTC*	Aerobic activated sludge bacterial community	EC_50_	36.3 mg L^−1^	[45]	1000	0.0363 ^c^	25,700–134,855	708–3715
DOX	*Lemna gibba*	EC_10_ (WW)	0.054 mg L^−1^	[46]	100	0.00054	175–2101	324–3890
	*Eisenia fetida*	EC_50_	96.1 mg kg^−1^	[16]	1000		96.1 ^b^	-

^a^ Data collected from Teixidó et al. [47]. The *K*_d_ range of each parent tetracycline was also applied to its corresponding TPs, as detailed in Appendix B Section Environmental Risk Assessment. ^b^ PNEC_soil_ calculated directly from terrestrial toxicity data; all other PNEC_soil_ values were estimated using the EqP method. ^c^ PNEC values for EOTC and ICTC were derived from limited experimental toxicity data. TPs are marked in italics. AF: assessment factor; EC_10_: 10% effective concentration; EC_50_: Median effective concentration; IR: inhibition rate; M: malformations; Ref.: Reference; RL: root length; WW: wet weight; -: no data.

**Table 3:** RQ of tetracyclines and their TPs in organic amendments.

		Manure								
		Horse			Poultry			Bovine		
Compound	PNEC_soil_ ^a^ (ng g^−1^)	Max ^b^ (ng g^−1^ dw)	PEC_soil_ _(_ng g^−1^_)_	RQ	Max ^b^ (ng g^−1^ dw)	PEC_soil_ _(_ng g^−1^_)_	RQ	Max ^b^ (ng g^−1^ dw)	PEC_soil_ _(_ng g^−1^_)_	RQ
TC	56	27.3	7.23 × 10^−5^	1.3 × 10^−3^	107	2.83 × 10^−4^	5 × 10^−3^	29.4	7.78 × 10^−5^	1.4 × 10^−3^
*ETC*	2484	38.9	1.03 × 10^−4^	4 × 10^−5^	95.4	2.53 × 10^−4^	1.0 × 10^−4^	4.1	1.09 × 10^−5^	4 × 10^−6^
OTC	460	113	2.99 × 10^−4^	7 × 10^−4^	21.1	5.59 × 10^−5^	1.2 × 10^−4^	83.4	2.21 × 10^−4^	5 × 10^−4^
*EOTC*	31	617	1.63 × 10^−3^	5 × 10^−2^	713	1.89 × 10^−3^	6 × 10^−2^	1069	2.83 × 10^−3^	9 × 10^−2^
CTC	44	15.5	4.10 × 10^−5^	9 × 10^−4^	-	-	-	17.2	4.55 × 10^−5^	1.0 × 10^−3^
*ICTC*	25,700	42.4	1.12 × 10^−4^	4 × 10^−6^	-	-	-	44.5	1.18 × 10^−4^	5 × 10^−6^
DOX	96.1	2161	5.72 × 10^−3^	6 × 10^−2^	402	1.06 × 10^−3^	1.1 × 10^−2^	2838	7.51 × 10^−3^	8 × 10^−2^
ΣRQ				9 × 10^−2^			7 × 10^−2^			1.4× 10^−1^
		**Sludge**								
		**Anaerobically Digested Sludge**			**Dehydrated Sludge**			**Composted Sludge**		
**Compound**	**PNEC_soil_ ^a^** **(ng g^−1^)**	**Max ^b^(ng g^−1^ dw)**	**PEC_soil_** **_(_** **ng g^−1^_)_**	**RQ**	**Max ^b^ (ng g^−1^ dw)**	**PEC_soil_** **_(_** **ng g^−1^_)_**	**RQ**	**Max ^b^ (ng g^−1^ dw)**	**PEC_soil_** **_(_** **ng g^−1^_)_**	**RQ**
TC	56	1238	1.82 × 10^−3^	3 × 10^−2^	590	8.68 × 10^−4^	2 × 10^−2^	11.5	1.69 × 10^−5^	3 × 10^−4^
*ETC*	2484	1113	1.64 × 10^−3^	7 × 10^−4^	459	6.75 × 10^−4^	3 × 10^−4^	5.47	8.04 × 10^−6^	3 × 10^−6^
*ATC*	1758	291	4.28 × 10^−4^	2 × 10^−4^	245	3.60 × 10^−4^	2 × 10^−4^	-	-	-
*EATC*	2528	119	1.75 × 10^−4^	7 × 10^−5^	94.7	1.39× 10^−4^	6 × 10^−5^	-	-	-
OTC	460	50.9	7.49 × 10^−5^	2 × 10^−4^	36.4	5.35 × 10^−5^	1.2 × 10^−4^	-	-	-
*EOTC*	31	1334	1.96 × 10^−3^	6 × 10^−2^	1002	1.47 × 10^−3^	5 × 10^−2^	80.6	1.19 × 10^−4^	4 × 10^−3^
CTC	44	10.4	1.53 × 10^−5^	4 × 10^−4^	10.2	1.50 × 10^−5^	3 × 10^−4^	-	-	-
DOX	96.1	2892	4.25 × 10^−3^	4 × 10^−2^	365	5.37× 10^−4^	6 × 10^−3^	-	-	-
ΣRQ				1.2 × 10^−1^			7 × 10^−2^			4 × 10^−3^

^a^ The lowest PNEC_soil_ value, derived using the lowest K_d_, was selected as the most conservative estimate for RQ calculation. PNEC_soil-EqP_ values were used for all compounds except DOX for which a PNEC_soil_ calculated directly from a terrestrial toxicity endpoint was used. ^b^ Maximum concentration quantified in samples (ng g^−1^ dw). Cells highlighted in light orange indicate low environmental risk (0.01 ≤ RQ < 0.1), whereas unshaded cells indicate insignificant risk (RQ < 0.01). ΣRQ: cumulative risk quotient.

## Data Availability

All data generated for this study are contained within the article and Supplementary Materials.

## Supplementary Materials

The following supporting information can be downloaded at https://www.mdpi.com/article/10.3390/antibiotics15090865/s1, Figure S1: Relative distribution of tetracyclines and their transformation products in different livestock manures: (a) OTC and EOTC; (b) CTC, ECTC and ICTC; (c) TC, ETC, ATC and EATC; Figure S2. Relative distribution of tetracyclines and their transformation products during sludge treatment conditions; Table S1. Concentration ranges, occurrence (frequency of detection) and mean concentrations of antibiotics in sludge; Table S2. Ecotoxicological endpoints reported in the literature for aquatic and terrestrial species exposed to tetracyclines and their transformation products; Table S3: Physical-chemical properties of the target tetracyclines and transformation products; Table S4. Recovery (R%). matrix effect (ME%), accuracy (A%) and precision of the method expressed as relative standard deviation (RSD) for composted sludge. References [55,56,57,58,59,60,61,62,63,64,65,66,67,68,69,70,71,72,73,74,75,76,77,78,79,80,81,82,83,84,85,86,87,88,89,90,91,92,93,94,95,96,97,98,99,100,101,102,103,104,105,106] are only cited in the Supplementary Materials.

## Abbreviations

The following abbreviations are used in this manuscript:

APPL_amendment_	application rate of organic amendments to soil
ATC	anhydrotetracycline
C_amendment_	concentration measured in organic amendment (dw)
CTC	chlortetracycline
DEPTH_soil_	mixing depth in soil
DMC	demeclocycline
dMRM	dynamic multiple reaction monitoring
DOX	doxycycline
dw	dry weight
EATC	4-epianhydrotetracycline
ECTC	4-epichlortetracycline
EC_10_	10% effective concentration
EC_50_	median effective concentration
EOTC	4-epioxytetracycline
EqP	equilibrium partitioning
ERA	Environmental Risk Assessment
ESI	electrospray ionization
ETC	4-epitetracycline
ICTC	isochlortetracycline
*K*_d_	soil-water distribution coefficient
LC-MS/MS	liquid chromatography coupled to tandem mass spectrometry
MDL	method detection limit
MSPD	matrix solid-phase dispersion
MQL	method quantification limit
NOEC	no observed effect concentration
MNC	minocycline
MTC	methacycline
OTC	oxytetracycline
PEC_soil_	predicted environmental concentration in the soil
PNEC_soil_	predicted no-effect concentration in the soil
QqQ	triple quadrupole mass spectrometer
RHO_soil_	bulk density of wet soil
RQ	risk quotient
SPE	solid-phase extraction
TC	tetracycline
WWTPs	wastewater treatment plants

## Appendix A. Analytical Method

### Sample Treatment and Analysis

The extraction of target compounds was carried out by MSPD using an empty 6-mL polypropylene solid-phase extraction (SPE) cartridge. Previously, freeze-dried sludge or manure sample (0.1 g dw) was mixed with silica gel as dispersant sorbent (0.4 g) using a laboratory mini-mill (Pulverisette 23, Fritsch, Idar-Oberstein, Germany) at 30 Hz for 2 min. The cartridges were prepared as follows: first, a filter frit was placed at the bottom of the cartridge and Florisil (0.8 g) and PSA (0.8 g) sorbents were added to perform the clean-up step. The cartridge was briefly vortex-mixed to obtain a homogeneous clean-up sorbent layer. A syringe plunger was placed on top, to prevent sorbent loss during vortex-mixing. Then, another frit was placed over the Florisil/PSA layer and the sample-dispersant mixture was added. A frit was placed at the top and the cartridge content was compacted using a syringe plunger. Target compounds were eluted from the cartridge by adding three consecutive 4 mL aliquots of a mixture of acetone (ACE) and Na_2_EDTA-McIlvaine buffer (pH 4) (30:70, *v*/*v*) using a vacuum manifold system (Waters, Milford, MA, USA) connected to a vacuum pump. The eluates were evaporated using an SC-200 Series Sample Concentrator equipped with a Stuart BH200 dry-block heater (Cole-Parmer^®^, Vernon Hills, IL, USA) under a gentle stream of nitrogen for 30 min. The remaining aqueous extract was transferred to a 10-mL volumetric flask and 50 μL of an IS aqueous solution (4 μg mL^−1^) was added to monitor potential volume fluctuations and injection repeatability during online SPE. The solution was diluted to the mark with pure water, filtered through a 0.2 μm regenerated cellulose syringe filter and collected in an autosampler vial for online SPE-LC-MS/MS determination. Analytical determination was performed using an Agilent 1290 Infinity II online SPE–LC system coupled to an Agilent 6495 triple quadrupole (QqQ) mass spectrometer (Agilent Technologies, Santa Clara, CA, USA) equipped with a vacuum degasser, a binary pump, and an automatic injector. The online SPE module consisted of a 900-μL loop, a quaternary pump, and a two-position 10-port switching valve. The switching valve allowed two SPE cartridges to operate simultaneously: one in the loading/conditioning position and the other in the elution position. A 100-μL aliquot of the MSPD sample extract was loaded into the online SPE loop. Then, the extract was transferred to a reversed-phase Bond Elut Online PLRP-S cartridge by passing water containing 2 mM ammonium formate and adjusted to pH 7.0 with ammonia solution at a flow rate of 1.5 mL min^−1^ for 2 min. After sample loading, the switching valve was moved to the elution position to transfer retained compounds onto the analytical column by the flow of the mobile phase. While online SPE cartridge 1 was being eluted, the previously used online SPE cartridge 2 was washed with ACE at a flow rate of 2.0 mL min^−1^ for 4.4 min and then conditioned with pH 7.0 ammonium formate buffer, to prepare it for the next sample. The LC system featured an autosampler with a 100-μL loop and a binary pump. Separation was achieved on a Zorbax RRHD Eclipse Plus C18 column (150 mm × 3.0 mm i.d., 1.8 μm particle size, Agilent Technologies, Santa Clara, CA, USA) thermostated at 35 °C and protected with a Zorbax RRHD Eclipse Plus C18 guard column (3.0 mm i.d., 1.8 μm particle size, Agilent Technologies, Santa Clara, CA, USA). The mobile phase consisted of 10 mM ammonium formate buffer, containing formic acid (0.1%, *v*/*v*) and 0.5 mM ammonium fluoride (solvent A) and MeOH (solvent B). Gradient elution was performed at 0.3 mL min^−1^ following this program: 10% solvent B was maintained for 5 min, then increased linearly to 20% over 1 min and held for 3 min. The proportion of solvent B was then increased to 40% in 1 min and held for 3 min. A linear increase to 45% was performed in 1 min, followed by a further increase to 60% over 3 min. Solvent B was then increased to 85% in 2 min and held for 3 min. Re-equilibration was achieved by decreasing solvent B linearly to 10% in 1 min and holding for 2 min, resulting in a total run time of 25 min. The mass spectrometer was equipped with an electrospray ionisation (ESI) source operating in positive mode. MS parameters were set as follows: capillary voltage, 4000 V; nebulizer pressure, 40 psi; gas temperature, 350 °C; drying gas flow rate, 11 L min^−1^; sheath gas temperature, 250 °C; sheath gas flow rate, 12 L min^−1^; and fragmentor voltage, 166 V. MS/MS analysis was conducted in Dynamic Multiple Reaction Monitoring mode. The two most abundant transitions were monitored for each analyte. The most abundant transition was used for quantification, whereas the less abundant transition was used for confirmation. Detailed LC–MS/MS parameters for each compound are provided in Table A1. Instrument control and data acquisition were managed using MassHunter Quantitative Analysis 10.1 software (Agilent Technologies, Santa Clara, CA, USA).

**Table A1:** LC-MS/MS conditions and retention times for the target compounds.

Compound	Precursor Ion (*m*/*z*)	Product Ions (Quantifier/ Qualifier) (*m*/*z*)	CE (eV)	Ion Ratio	Retention Time (min)
TC	445.2	410.2; 154.1	20; 30	71.1	14.23
*ETC*	445.1	410.2; 98.1	20; 44	44.3	13.36
*ATC*	427.2	153.9; 410.1	20; 28	221.6	21.71
*EATC*	427.2	410.1; 321.1	20; 32	11.0	19.40
OTC	461.2	426.1; 443.0	20; 12	47.3	14.56
*EOTC*	461.2	426.2; 444.0	20; 16	73.9	13.92
CTC	479.1	444.0; 462.0	20; 20	66.0	17.76
*ECTC*	479.0	444.0; 98.0	20; 42	82.2	16.21
*ICTC*	479.1	462.1; 196.8	20; 45	20.6	14.49
MTC	443.0	426.0; 381.0	25; 25	33.7	18.19
DOX	445.2	428.0; 154.0	30; 30	41.0	19.07
MNC	458.2	441.1; 283.1	20; 48	20.4	16.83
DMC	465.1	448.1; 430.1	16; 24	61.6	15.85

CE: collision energy. TPs are marked in italics.

## Appendix B. Environmental Risk Assessment

### Selection of Ecotoxicological Endpoints and Derivation of PNEC Values

All ecotoxicological endpoints were selected according to the EMA guideline [51], prioritizing chronic (EC_10_ or NOEC) over acute toxicity data (EC_50_ or LC_50_) and EC_10_ over NOEC. When both terrestrial and aquatic ecotoxicological data were available for a given compound, PNEC_soil_ values derived from both approaches were calculated and compared. Corresponding RQ values were subsequently calculated using the most sensitive PNEC_soil_ value, thereby representing a worst-case risk assessment scenario.

Aquatic ecotoxicological data were obtained from primary producers (algae), primary consumers (e.g., *Daphnia magna*), and secondary consumers (fish). Additionally, some toxicity data for terrestrial plants (e.g., carrot, pepper, lettuce, onion, radish, and ryegrass) were generated under aqueous exposure conditions, typically based on seed germination and plant growth endpoints (root, shoot, leaf, or stem growth). Therefore, these data were considered equivalent to aquatic toxicity data for the purpose of deriving PNEC_water_ values and subsequent PNEC_soil_ estimates using the EqP approach.

Terrestrial ecotoxicological data were obtained from soil-dwelling invertebrates, including earthworms, enchytraeids, and springtails, using survival, growth, and reproduction endpoints, as well as from terrestrial plants exposed in soil (e.g., cucumber and lettuce), using germination, growth, and root elongation endpoints. This approach assumes that toxicity to soil organisms is driven by the concentration in soil pore water and provides a consistent framework to extrapolate aquatic toxicity data to soil conditions. This is consistent with current European regulatory approaches for linking aquatic and terrestrial compartments [51].

Moreover, bacterial endpoints were only considered for ICTC and EOTC, for which just one experimental ecotoxicological endpoint was found in the scientific literature. Although additional predicted toxicity values were available from [6,7,53], experimentally derived ecotoxicological data were prioritized whenever available.

The annual application rates used for PEC_soil_ estimation were selected according to European guidance and previous studies as representative scenarios. For sewage sludge, an application rate of 0.5 kg m^−2^ yr^−1^ was used, corresponding to the application rate assumed in the European Commission Technical Guidance Document for sludge applied to agricultural soil [52]. For manure, an application rate of 0.9 kg m^−2^ yr^−1^ was selected as a representative agricultural application rate based on previous studies. Li et al. [49] considered an annual application of 6000 kg of dry manure to 0.67 ha, corresponding to approximately 9 t ha^−1^ yr^−1^. The same application rate was also used by Wu et al. [7]. In addition, Ghirardini et al. [18] considered a manure application scenario of 9.5 t ha^−1^ yr^−1^.

To ensure a conservative risk assessment, the lowest *K*_d_ values reported by Teixidó et al. [47] were selected. Since lower *K*_d_ values produce lower EqP-derived PNEC_soil_ values, this approach yields higher RQ estimates and represents a worst-case scenario. Soil-water distribution coefficients (*K*_d_) for tetracycline antibiotics are strongly influenced by compound speciation and soil properties, including pH, cation exchange capacity, metal oxide content, and texture [47,107]. Figueroa-Diva et al. [107] reported similar *K*_d_ values for TC, OTC, and CTC within each soil–pH pair and concluded that R-group substituents have little influence on tetracycline sorption, suggesting that *K*_d_ values measured for representative compounds may be used to approximate the sorption of structurally related members of the same family [107]. In the absence of experimental *K*_d_ data for the TPs and given the close structural similarity between the parent tetracyclines and their corresponding TPs, particularly in the case of epimeric TPs, considered in this study, the *K*_d_ range reported for each parent tetracycline was therefore applied to its TPs as an approximation for the calculation of EqP-derived PNEC_soil_ values.
