# Supplementary material for: Occurrence and Risk Assessment of Tetracyclines and Their Transformation Products in Organic Amendments
Source: Antibiotics (Basel). 2026 Sep 4;15(9):865. doi: 10.3390/antibiotics15090865 (PMC13603315; doi:10.3390/antibiotics15090865)
Supplement: Supplementary file 1 [file antibiotics-15-00865-s001.zip › antibiotics-4516669-supplementary.pdf]

Supplementary material

Occurrence and risk assessment of tetracyclines and their transformation products in organic amendments

Noelia García-Criado, Juan Luis Santos, Julia Martín, Irene Aparicio, Esteban Alonso*

Departamento de Química Analítica, Escuela Politécnica Superior-CATEPS, Universidad de Sevilla. C/ Euclides, s/n, E-41092 Seville, Spain

---

*Corresponding autor

E-mail address: ealonso@us.es

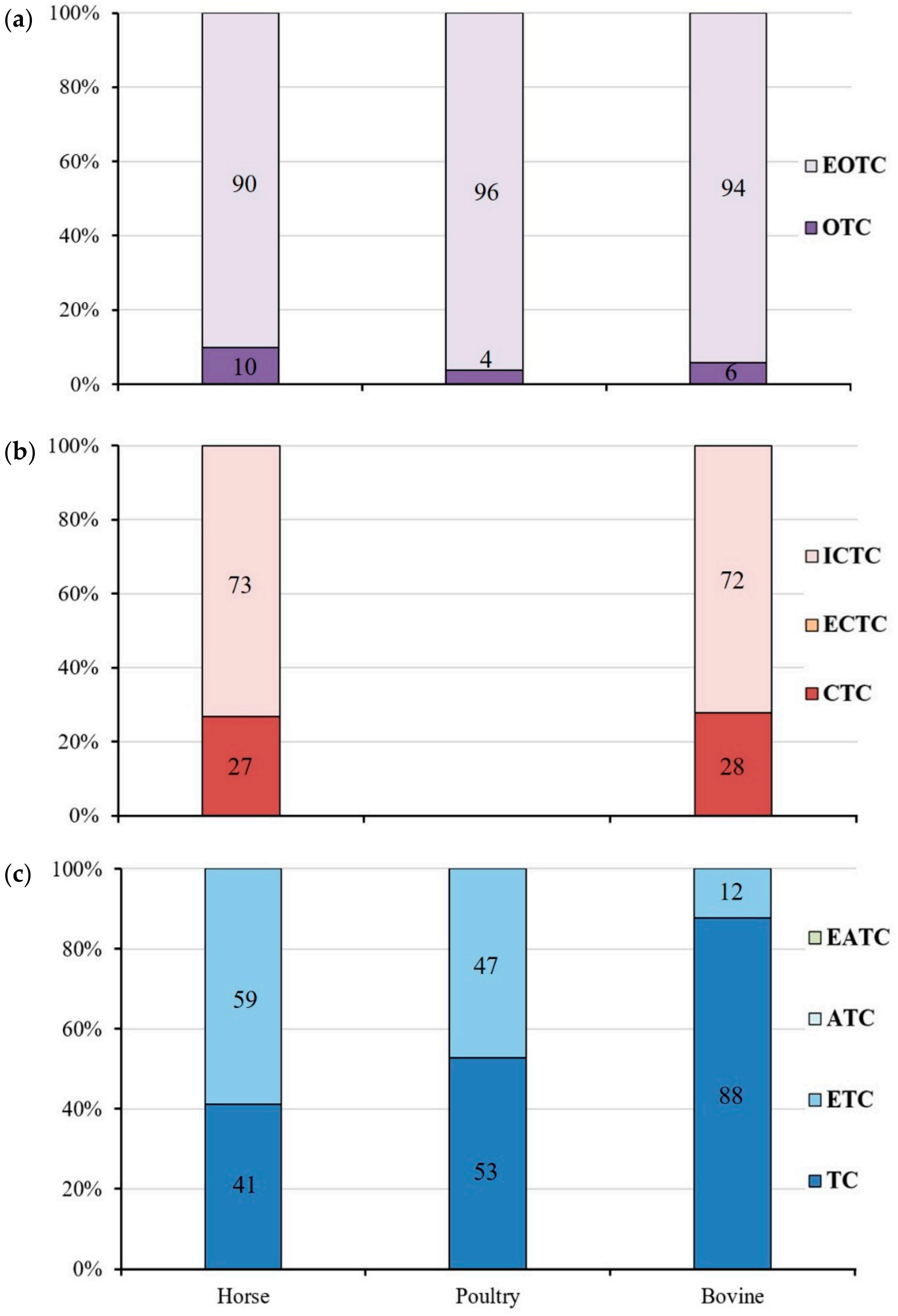

Figure S1. Relative distribution of tetracyclines and their transformation products (TPs) in different livestock manures: (a) OTC and EOTC; (b) CTC, ECTC and ICTC; (c) TC, ETC, ATC and EATC.

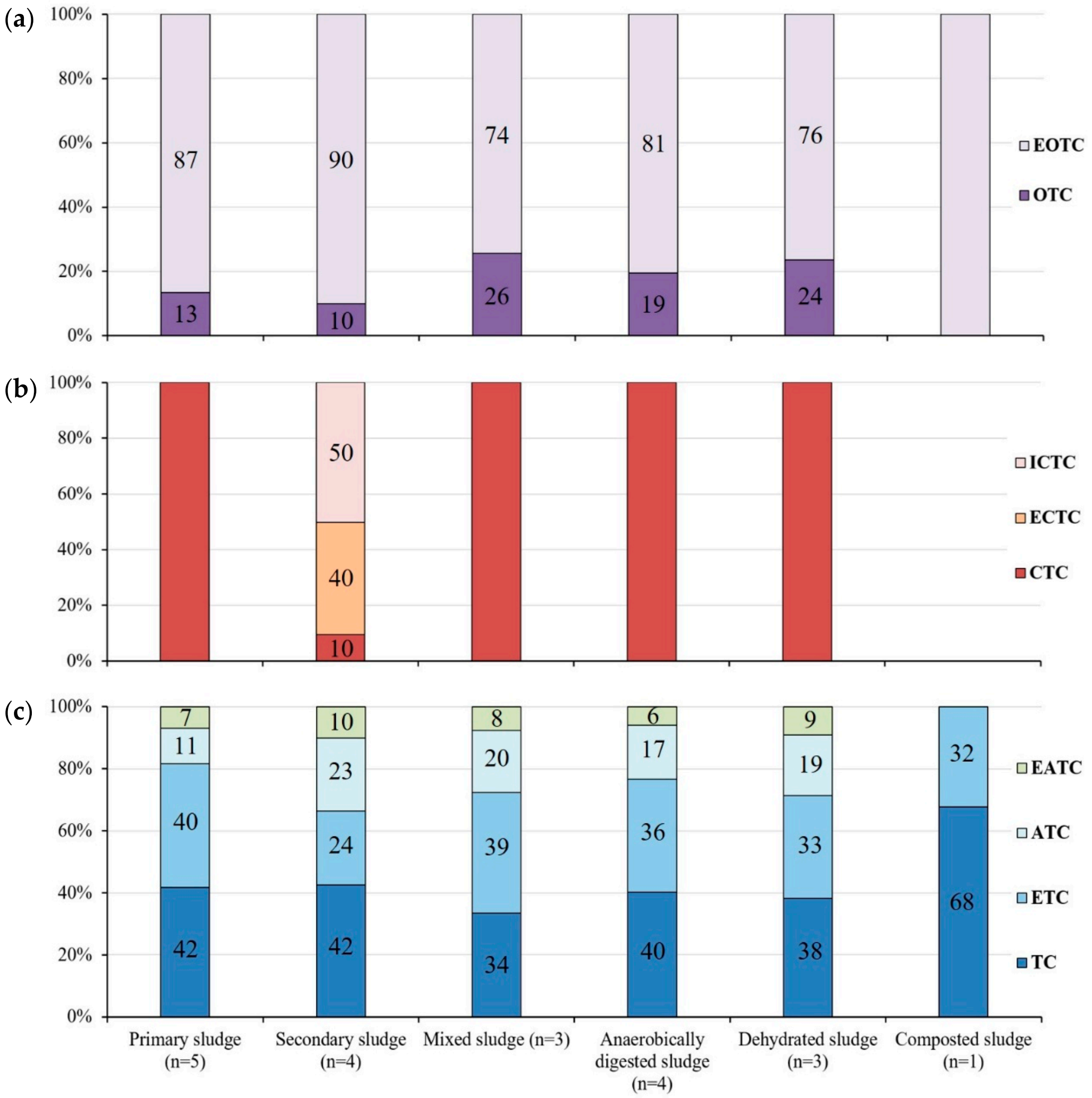

Figure S2. Relative distribution of tetracyclines and their TPs during sludge treatment conditions: (a) OTC and EOTC; (b) CTC, ECTC and ICTC; (c) TC, ETC, ATC and EATC.

Table S1. Concentration ranges, occurrence (frequency of detection) and mean concentrations of antibiotics in sludge.

Compound	Sludge Concentration																	
	**Primary sludge (n=5)**			**Secondary sludge (n=4)**			**Mixed sludge (n=3)**			**Anaerobically digested sludge (n=4)**			**Dehydrated sludge (n=3)**			**Composted sludge (n=1)**		
	Mean (ng g^{-1})	Range (ng g^{-1})	Occ. (%)	Mean (ng g^{-1})	Range (ng g^{-1})	Occ. (%)	Mean (ng g^{-1})	Range (ng g^{-1})	Occ. (%)	Mean (ng g^{-1})	Range (ng g^{-1})	Occ. (%)	Mean (ng g^{-1})	Range (ng g^{-1})	Occ. (%)	Mean (ng g^{-1})	Range (ng g^{-1})	Occ. (%)
TC	543	44.2-1091	100	338	<MQL-486	75	304	55.7-550	100	638	432-1238	100	304	128-590	100	11.5	-	100
ETC	520	<MDL-979	80	190	137-289	100	353	<MDL-416	67	579	336-1113	100	263	127-459	100	5.47	-	100
ATC	148	<MDL-190	40	187	<MQL-227	50	181	<MDL-181	33	275	<MQL-291	50	155	98.0-245	100	-	-	0
EATC	90.5	<MQL-119	80	80.6	<MDL-93.3	75	69.1	59.6-86.5	100	95.1	70.0-119	100	72.6	57.1-94.7	100	-	-	0
OTC	28.3	<MDL-36.2	80	30.4	<MDL-34.6	75	24.4	23.1-26.9	100	37.3	21.4-50.9	100	27.9	22.3-36.4	100	-	-	0
EOTC	181	155-541	100	275	<MQL-362	75	70.6	194-845	100	155	217-1334	100	90.5	329-1002	100	80.6	-	100
CTC	5.55	<MDL-10.5	80	7.1	<MDL-7.1	25	10.3	<MDL-10.5	67	7.57	<MDL-10.4	75	5.75	<MDL-10.2	67	-	-	0
ECTC	-	-	0	29.7	<MDL-29.7	25	-	-	0	-	-	0	-	-	0	-	-	0
ICTC	-	-	0	37.0	<MDL-37.0	25	-	-	0	-	-	0	-	-	0	-	-	0
DOX	859	74.2-1534	100	656	224-1127	100	290	<MQL-379	67	1581	859-2892	100	390	<MQL-365	67	-	-	0
MTC	-	-	0	-	-	0	-	-	0	-	-	0	-	-	0	-	-	0
MNC	-	-	0	206	<MDL-216	50	-	-	0	-	-	0	-	-	0	-	-	0

Mean concentrations of quantified samples; Concentrations are reported as ng g^{-1} dry weight (dw). TPs are marked in italics. Occ.: Occurrence; -: <MDL

Table S2. Ecotoxicological endpoints reported in the literature for aquatic and terrestrial species exposed to tetracyclines and their transformation products.

Cmpd.	Trophic level	Phylum/organism group	Species (common name)	Time (days)	Endpoint (effect assessed)	Effect (mg L^{-1})	Reference
TC	Primary producers	Cyanobacteria	*Microcystis aeruginosa*	4	EC_{50}	0.09	(Halling-Sørensen, 2000) [55]
	Primary producers	Cyanobacteria	*Microcystis aeruginosa*	4	EC_{10}	0.63	(Ye et al., 2017) [56]
	Primary producers	Cyanobacteria	*Microcystis aeruginosa*	4	EC_{20}	1.58	(Ye et al., 2017) [56]
	Primary producers	Cyanobacteria	*Anabaena flos-aquae*	3	EC_{50}	2.7	(Kolar et al., 2014) [57]
	Primary producers	Cyanobacteria	*Anabaena flos-aquae*	3	EC_{10}	1.5	(Kolar et al., 2014) [57]
	Primary producers	Cyanobacteria	*Anabaena* sp. CPB4337	3	EC_{10}	2.5	(González-Pleiter et al., 2013) [58]
	Primary producers	Green algae	*Pseudokirchneriella subcapitata*	3	EC_{50}	3.31	(González-Pleiter et al., 2013) [58]
	Primary producers	Green algae	*Pseudokirchneriella subcapitata*	3	EC_{10}	0.032	(González-Pleiter et al., 2013) [58]
	Primary producers	Aquatic plant	*Lemna gibba*	7	EC_{50}	0.723	(Brain et al., 2004) [46]
	Primary producers	Aquatic plant	*Lemna minor*	7	EC_{50}	1.06	(Pomati et al., 2004) [59]
	Primary producers	Green algae	*Pseudokirchneriella subcapitata*	4	EC_{50}	2.2	(Halling-Sørensen, 2000) [55]
	Primary producers	Green algae	*Pseudokirchneriella subcapitata*	3	EC_{50}	1.0	(Yang et al., 2008) [60]
	Primary producers	Green algae	*Pseudokirchneriella subcapitata*	3	NOEC	0.5	(Yang et al., 2008) [60]
	Primary producers	Green algae	*Pseudokirchneriella subcapitata*	3	LOEC	1.0	(Yang et al., 2008) [60]
	Primary producers	Green algae	*Raphidocelis subcapitata*	3	EC_{50}	0.58	(Kovalakova et al., 2021) [61]
	Primary producers	Green algae	*Raphidocelis subcapitata*	3	NOEC	0.31	(Kovalakova et al., 2021) [61]
	Primary producers	Green algae	*Raphidocelis subcapitata*	3	LOEC	0.63	(Kovalakova et al., 2021) [61]
	Primary producers	Green algae	*Scenedesmus obliquus*	4	IC_{10}	1.8	(Chen et al., 2022) [62]
	Primary producers	Green algae	*Scenedesmus obliquus*	8	IC_{50}	6.9	(Chen et al., 2022) [62]
	Primary producers	Green algae	*Chlorella vulgaris*	4	EC_{50}	7.73	(Xu et al., 2019) [41]
	Primary producers	Terrestrial plant (dicot)	*Daucus carota* (carrot)	7	EC_{50}	0.212	(Hillis et al., 2011) [39]
	Primary producers	Terrestrial plant (dicot)	*Daucus carota* (carrot)	7	EC_{10}	0.0019	(Hillis et al., 2011) [39]
	Primary producers	Terrestrial plant (dicot)	*Daucus carota* (carrot)	7	LOEC	0.100	(Hillis et al., 2011) [39]
	Primary producers	Terrestrial plant (dicot)	*Lactuca sativa* (lettuce)	5	EC_{10}	1.433	(Hillis et al., 2011) [39]
	Primary producers	Terrestrial plant (dicot)	*Lactuca sativa* (lettuce)	5	EC_{50}	>250	(Pino et al., 2016) [63]

Cmpd.	Trophic level	Phylum/organism group	Species (common name)	Time (days)	Endpoint (effect assessed)	Effect (mg L^{-1})	Reference
	Primary producers	Terrestrial plant (dicot)	*Lactuca sativa* (lettuce)	5	EC_{50}	453	(Pino et al., 2016) [63]
	Primary producers	Terrestrial plant (dicot)	*Medicago sativa* (alfalfa)	5	EC_{10}	0.0071	(Hillis et al., 2011) [39]
TC	Primary producers	Terrestrial plant (dicot)	*Lactuca sativa (*lettuce)	5	NOEC	0.01	(Pan et al., 2016) [64]
	Primary producers	Terrestrial plant (dicot)	*Lactuca sativa (*lettuce)	5	LOEC	0.1	(Pan and Chu., 2017) [65]
	Primary producers	Terrestrial plant (dicot)	*Lactuca sativa (*lettuce)	5	EC_{10}	0.11	(Pan et al., 2016) [64]
	Primary producers	Terrestrial plant (dicot)	*Lactuca sativa (*lettuce)	5	EC_{50}	14.4	(Pan et al., 2016) [64]
	Primary producers	Terrestrial plant (dicot)	*Daucus carota* (carrot)	7	NOEC	<0.01	(Pan et al., 2016) [64]
	Primary producers	Terrestrial plant (dicot)	*Daucus carota* (carrot)	7	LOEC	0.01	(Pan et al., 2016) [64]
	Primary producers	Terrestrial plant (dicot)	*Daucus carota* (carrot)	7	EC_{10}	0.26	(Pan et al., 2016) [64]
	Primary producers	Terrestrial plant (dicot)	*Daucus carota* (carrot)	7	EC_{50}	10.3	(Pan et al., 2016) [64]
	Primary producers	Terrestrial plant (dicot)	*Cucumis sativus* L. (cucumber)	7	NOEC	0.1	(Pan et al., 2016) [64]
	Primary producers	Terrestrial plant (dicot)	*Cucumis sativus* L. (cucumber)	7	LOEC	1	(Pan et al., 2016) [64]
	Primary producers	Terrestrial plant (dicot)	*Cucumis sativus* L. (cucumber)	7	EC_{10}	0.43	(Pan et al., 2016) [64]
	Primary producers	Terrestrial plant (dicot)	*Cucumis sativus* L. (cucumber)	7	EC_{50}	24.8	(Pan et al., 2016) [64]
	Primary producers	Terrestrial plant (dicot)	Tomato	5	NOEC	<0.01	(Pan et al., 2016) [64]
	Primary producers	Terrestrial plant (dicot)	Tomato	5	LOEC	0.01	(Pan et al., 2016) [64]
	Primary producers	Terrestrial plant (dicot)	Tomato	5	EC_{10}	0.10	(Pan et al., 2016) [64]
	Primary producers	Terrestrial plant (dicot)	Tomato	5	EC_{50}	11.6	(Pan et al., 2016) [64]
	Primary producers	Terrestrial plant (monocot)	*Triticum aestivum* L. (wheat)	3	IC_{50}	177	(Xie et al., 2011) [66]
	Primary producers	Terrestrial plant (dicot)	*Sinapis alba* L.	5	EC_{50}	109	(Timmerer et al., 2020) [67]
	Primary producers	Terrestrial plant (dicot)	*Cichorium endivia* (endive)	4-5	EC_{50}	57	(Liu et al., 2009) [40]
	Primary producers	Terrestrial plant (dicot)	*Cichorium endivia* (endive)	4-5	EC_{10}	14	(Liu et al., 2009) [40]
	Primary producers	Terrestrial plant (monocot)	Rice	4-5	EC_{50}	69	(Liu et al., 2009) [40]
	Primary producers	Terrestrial plant (monocot)	Rice	4-5	EC_{10}	16	(Liu et al., 2009) [40]
	Primary producers	Terrestrial plant (dicot)	*Cucumis sativus L.* (cucumber)	4-5	EC_{50}	203	(Liu et al., 2009) [40]
	Primary producers	Terrestrial plant (dicot)	*Cucumis sativus L.* (cucumber)	4-5	EC_{10}	8	(Liu et al., 2009) [40]
	Primary consumers	Crustacean	*Daphnia magna*	2	EC_{50}	198	(Sönmez and Sivri, 2020) [68]
	Primary consumers	Crustacean	*Daphnia magna*	21	EC_{50}	44.8	(Wollenberger et al., 2000) [69]
	Primary consumers	Crustacean	*Daphnia magna*	21	EC_{10}	29.4	(Wollenberger et al., 2000) [69]
	Secondary consumers	Planarian	*Dugesia japonica*	1	LC_{50}	1000	(Li, 2013) [70]

Cmpd.	Trophic level	Phylum/organism group	Species (common name)	Time (days)	Endpoint (effect assessed)	Effect (mg L^{-1})	Reference
	Secondary consumers	Planarian	*Dugesia japonica*	2	LC_{50}	760.6	(Li, 2013) [70]
	Secondary consumers	Planarian	*Dugesia japonica*	3	LC_{50}	298.1	(Li, 2013) [70]
TC	Secondary consumers	Planarian	*Dugesia japonica*	4	LC_{50}	206.4	(Li, 2013) [70]
	Secondary consumers	Fish	*Salvelinus namaycush*	4	LC_{50}	220	(Marking et al., 1988) [71]
	Secondary consumers	Fish	*Gambusia holbrooki*	4	LOEC	<0.000005	(Nunes et al., 2015) [72]
	Secondary consumers	Fish	*Danio rerio* embryos	4	EC_{10}	0.00316	(Q. Zhang et al., 2015) [73]
	Secondary consumers	Fish	*Danio rerio* embryos	4	LC_{10}	552	(Q. Zhang et al., 2015) [73]
	Secondary consumers	Fish	*Danio rerio* embryos	4	LC_{50}	760	(Q. Zhang et al., 2015) [73]
	Secondary consumers	Fish	*Gambusia holbrooki*	4	NOEC	0.000005	(Q. Zhang et al., 2015) [73]
					HC_5	0.00000874	(Q. Zhang et al., 2015) [73]
	Primary producers	Terrestrial plant	*Cucumis sativus* L. (cucumber)	22	EC_{10}	300 mg kg^{-1}	(Liu et al., 2009) [39]
	Primary consumer	Annelida	*Eisenia fetida* (earthworm)	14	LC_{50}	>2000 mg kg^{-1}	(Pino et al., 2015) [74]
ETC	Primary producers	Green algae	*Chlorella vulgaris*	4	EC_{50}	8.42	(Xu et al., 2019) [40]
ATC	Primary producers	Green algae	*Chlorella vulgaris*	4	EC_{50}	5.96	(Xu et al., 2019) [40]
EATC	Secondary consumers	Fish	*Danio rerio* embryos	4	LC_{50}	29.13	(Wang et al., 2020) [41]
	Secondary consumers	Fish	*Danio rerio* embryos	4	EC_{50}	8.57	(Wang et al., 2020) [41]
OTC	Primary producers	Cyanobacteria	*Microcystis aeruginosa*	7	EC_{50}	0.207	(Holten Lützhøft et al., 1999) [75]
	Primary producers	Cyanobacteria	*Microcystis aeruginosa*	1	EC_{50}	5.4	(van der Grinten et al., 2010) [76]
	Primary producers	Cyanobacteria	*Microcystis aeruginosa*	6	EC_{50}	0.23	(Ando et al., 2007) [77]
	Primary producers	Cyanobacteria	*Microcystis aeruginosa*	4	EC_{10}	3.02	(Ye et al., 2017) [56]
	Primary producers	Cyanobacteria	*Microcystis aeruginosa*	4	EC_{20}	4.86	(Ye et al., 2017) [56]
	Primary producers	Cyanobacteria	*Microcystis wesenbergii*	6	EC_{50}	0.35	(Ando et al., 2007) [77]
	Primary producers	Cyanobacteria	*Synechococcus leopoliensis*	6	EC_{50}	1.1	(Ando et al., 2007) [77]
	Primary producers	Cyanobacteria	*Synechococcus* sp.	6	EC_{50}	2	(Ando et al., 2007) [77]
	Primary producers	Cyanobacteria	*Anabaena cylindrica* NIES-19	6	EC_{50}	0.032	(Ando et al., 2007) [77]
	Primary producers	Cyanobacteria	*Anabaena cylindrica* NIES-19	6	NOEC	0.0031	(Ando et al., 2007) [77]
	Primary producers	Cyanobacteria	*Anabaena flos-aquae*	6	NOEC	0.025	(Ando et al., 2007) [77]
	Primary producers	Cyanobacteria	*Anabaena variabilis* NIES-23	6	NOEC	0.100	(Ando et al., 2007) [77]

Cmpd.	Trophic level	Phylum/organism group	Species (common name)	Time (days)	Endpoint (effect assessed)	Effect (mg L^{-1})	Reference
	Primary producers	Cyanobacteria	*Anabaena flos-aquae*	6	EC_{50}	0.39	(Ando et al., 2007) [77]
	Primary producers	Cyanobacteria	*Anabaena variabilis*	6	EC_{50}	0.36	(Ando et al., 2007) [77]
	Primary producers	Cyanobacteria	*Anabaena flos-aquae*	3	EC_{10}	1.5	(Kolar et al., 2014) [57]
	Primary producers	Cyanobacteria	*Anabaena flos-aquae*	3	EC_{50}	2.7	(Kolar et al., 2014) [57]
OTC	Primary producers	Diatoms	*Phaeodactylum tricornutum*	4	EC_{50}	1.73	(de Orte et al., 2013) [78]
	Primary producers	Green algae	*Scenedesmus vacuolatus*	1	EC_{50}	40.4	(Kołodziejska et al., 2013) [79]
	Primary producers	Green algae	*Pseudokirchneriella subcapitata*	3	EC_{50}	0.17	(Isidori et al., 2005) [80]
	Primary producers	Green algae	*Pseudokirchneriella subcapitata*	3	EC_{50}	4.18	(De Liguoro et al., 2003) [81]
	Primary producers	Green algae	*Pseudokirchneriella subcapitata*	4	EC_{50}	4.5	(Holten Lützhøft et al., 1999) [75]
	Primary producers	Green algae	*Pseudokirchneriella subcapitata*	4	IC_{50}	0.64	(Carusso et al., 2018) [82]
	Primary producers	Green algae	*Pseudokirchneriella subcapitata*	3	EC_{50}	0.342	(Eguchi et al., 2004) [83]
	Primary producers	Green algae	*Pseudokirchneriella subcapitata*	3	NOEC	0.183	(Eguchi et al., 2004) [83]
	Primary producers	Green algae	*Pseudokirchneriella subcapitata*	4	EC_{50}	3.1	(Zounková et al., 2011) [84]
	Primary producers	Green algae	*Pseudokirchneriella subcapitata*	4	NOEC	0.78	(Zounková et al., 2011) [84]
	Primary producers	Green algae	*Pseudokirchneriella subcapitata*	4	LOEC	1.6	(Zounková et al., 2011) [84]
	Primary producers	Green algae	*Pseudokirchneriella subcapitata*	3	EC_{50}	1.04	(Kolar et al., 2014) [57]
	Primary producers	Green algae	*Pseudokirchneriella subcapitata*	3	EC_{10}	0.47	(Kolar et al., 2014) [57]
	Primary producers	Green algae	*Pseudokirchneriella subcapitata*	1	EC_{50}	0.6	(van der Grinten et al., 2010) [76]
	Primary producers	Green algae	*Pseudokirchneriella subcapitata*	4	EC_{50}	1.61	(Fu et al., 2017) [85]
	Primary producers	Green algae	*Pseudokirchneriella subcapitata*	3	EC_{50}	2.55	(Fu et al., 2017) [85]
	Primary producers	Green algae	*Pseudokirchneriella subcapitata*	3	EC_{50}	1.04	(Kolar et al., 2014) [57]
	Primary producers	Green algae	*Raphidocelis subcapitata*	3	EC_{50}	2.05	(Kovalakova et al., 2021) [61]
	Primary producers	Green algae	*Raphidocelis subcapitata*	3	NOEC	0.16	(Kovalakova et al., 2021) [61]
	Primary producers	Green algae	*Raphidocelis subcapitata*	3	LOEC	0.31	(Kovalakova et al., 2021) [61]
	Primary producers	Green algae	*Chlorella vulgaris*	3	EC_{50}	7.05	(Eguchi et al., 2004) [83]
	Primary producers	Green algae	*Chlorella vulgaris*	3	NOEC	3.58	(Eguchi et al., 2004) [83]
	Primary producers	Green algae	*Ankistrodesmus fusiformis*	4	IC_{50}	4.17	(Carusso et al., 2018) [82]
	Primary producers	Green algae	*Isochrysis galbana*	4	EC_{50}	6.43	(de Orte et al., 2013) [78]
	Primary producers	Green algae	*Tetraselmis chuii*	4	IC_{50}	11.18	(Ferreira et al., 2007) [86]

Cmpd.	Trophic level	Phylum/organism group	Species (common name)	Time (days)	Endpoint (effect assessed)	Effect (mg L^{-1})	Reference
	Primary producers	Green algae	*Tetraselmis chuii*	3	IC_{50}	13.16	(Ferreira et al., 2007) [86]
	Primary producers	Green algae	*Tetraselmis suecica*	4	IC_{50}	17.25	(Seoane et al., 2014) [87]
	Primary producers	Cryptophyta	*Rhodomonas salina*	2	EC_{50}	1.6	(Holten Lützhøft et al., 1999) [75]
	Primary producers	Aquatic plant	*Lemna gibba*	7	EC_{50}	1.152	(Brain et al., 2004) [45]
	Primary producers	Aquatic plant	*Lemna minor*	7	EC_{50}	2.1	(Zounková et al., 2011) [84]
OTC	Primary producers	Aquatic plant	*Lemna minor*	7	NOEC	1	(Zounková et al., 2011) [84]
	Primary producers	Aquatic plant	*Lemna minor*	7	EC_{50}	3.26	(Kołodziejska et al., 2013) [79]
	Primary producers	Terrestrial plant (dicot)	*Daucus carota* (carrot)	**7**	EC_{50}	4.909	(Hillis et al., 2011) [39]
	Primary producers	Terrestrial plant (dicot)	*Daucus carota* (carrot)	**7**	EC_{10}	0.536	(Hillis et al., 2011) [39]
	Primary producers	Terrestrial plant (dicot)	*Daucus carota* (carrot)	60	LOEC	1.000	(Hillis et al., 2011) [39]
	Primary producers	Terrestrial plant (dicot)	*Lactuca sativa* (lettuce)	5	EC_{25}	4.781	(Hillis et al., 2011) [39]
	Primary producers	Terrestrial plant (dicot)	*Lactuca sativa* (lettuce)	5	EC_{10}	1.815	(Hillis et al., 2011) [39]
	Primary producers	Terrestrial plant (dicot)	*Medicago sativa* (alfalfa)	5	EC_{10}	6.730	(Hillis et al., 2011) [39]
	Primary producers	Terrestrial plant (monoc)	*Avena sativa* (oat)	3-11	EC_{50}	18.02	(Sacristán et al., 2025) [15]
	Primary producers	Terrestrial plant (monoc)	*Avena sativa* (oat)	3-11	EC_{10}	0.39	(Sacristán et al., 2025) [15]
	Primary producers	Terrestrial plant (monoc)	*Hordeum vulgare* (barley)	3-11	EC_{50}	556.62	(Sacristán et al., 2025) [15]
	Primary producers	Terrestrial plant (monoc)	*Hordeum vulgare* (barley)	3-11	EC_{10}	2.08	(Sacristán et al., 2025) [15]
	Primary producers	Terrestrial plant (monoc)	*Triticum aestivum* (wheat)	3-11	EC_{50}	466.32	(Sacristán et al., 2025) [15]
	Primary producers	Terrestrial plant (monoc)	*Triticum aestivum* (wheat)	3-11	EC_{10}	1.33	(Sacristán et al., 2025) [15]
	Primary producers	Terrestrial plant (monoc)	*Zea mays* (maize)	3-11	EC_{50}	846.78	(Sacristán et al., 2025) [15]
	Primary producers	Terrestrial plant (monoc)	*Zea mays* (maize)	3-11	EC_{10}	3.04	(Sacristán et al., 2025) [15]
	Primary producers	Terrestrial plant (dicot)	*Helianthus annuus* (sunflower)	3-11	EC_{50}	151.43	(Sacristán et al., 2025) [15]
	Primary producers	Terrestrial plant (dicot)	*Helianthus annuus* (sunflower)	3-11	EC_{10}	6.19	(Sacristán et al., 2025) [15]
	Primary producers	Terrestrial plant (dicot)	*Lactuca sativa* (lettuce)	3-11	EC_{50}	782.31	(Sacristán et al., 2025) [15]
	Primary producers	Terrestrial plant (dicot)	*Lactuca sativa* (lettuce)	3-11	EC_{10}	26.64	(Sacristán et al., 2025) [15]
	Primary producers	Terrestrial plant (dicot)	*Brassica napus* (rapeseed)	3-11	EC_{50}	54.57	(Sacristán et al., 2025) [15]
	Primary producers	Terrestrial plant (dicot)	*Brassica napus* (rapeseed)	3-11	EC_{10}	0.50	(Sacristán et al., 2025) [15]
	Primary producers	Terrestrial plant (dicot)	*Brassica oleracea* (cabbage)	3-11	EC_{50}	45.98	(Sacristán et al., 2025) [15]
	Primary producers	Terrestrial plant (dicot)	*Brassica oleracea* (cabbage)	3-11	EC_{10}	1.36	(Sacristán et al., 2025) [15]

Cmpd.	Trophic level	Phylum/organism group	Species (common name)	Time (days)	Endpoint (effect assessed)	Effect (mg L^{-1})	Reference
	Primary producers	Terrestrial plant (dicot)	*Vicia sativa* (common vetch)	3-11	EC_{50}	289.01	(Sacristán et al., 2025) [15]
	Primary producers	Terrestrial plant (dicot)	*Vicia sativa* (common vetch)	3-11	EC_{10}	9.14	(Sacristán et al., 2025) [15]
	Primary producers	Terrestrial plant (monoc)	*Avena sterilis* (sterile oat)	3-11	NOEC	1	(Sacristán et al., 2025) [15]
	Primary producers	Terrestrial plant (dicot)	*Bellis perennis* (english daisy)	3-11	EC_{50}	331.48	(Sacristán et al., 2025) [15]
	Primary producers	Terrestrial plant (dicot)	*Bellis perennis* (english daisy)	3-11	EC_{10}	17.82	(Sacristán et al., 2025) [15]
	Primary producers	Terrestrial plant (dicot)	*Centaurea cyanus* (cornflower)	3-11	EC_{50}	1449.49	(Sacristán et al., 2025) [15]
OTC	Primary producers	Terrestrial plant (dicot)	*Centaurea cyanus* (cornflower)	3-11	EC_{10}	0.18	(Sacristán et al., 2025) [15]
	Primary producers	Terrestrial plant (dicot)	*Lotus corniculatus* (birdsfoot trefoil)	3-11	EC_{50}	178.84	(Sacristán et al., 2025) [15]
	Primary producers	Terrestrial plant (dicot)	*Lotus corniculatus* (birdsfoot trefoil)	3-11	EC_{10}	2.38	(Sacristán et al., 2025) [15]
	Primary producers	Terrestrial plant (dicot)	*Trifolium pratense* (red clover)	3-11	EC_{50}	2611.49	(Sacristán et al., 2025) [15]
	Primary producers	Terrestrial plant (dicot)	*Trifolium pratense* (red clover)	3-11	EC_{10}	64.34	(Sacristán et al., 2025) [15]
	Primary producers	Terrestrial plant (dicot)	*Digitalis purpurea* (purple foxglove)	3-11	EC_{50}	63.98	(Sacristán et al., 2025) [15]
	Primary producers	Terrestrial plant (dicot)	*Digitalis purpurea* (purple foxglove)	3-11	EC_{10}	21.59	(Sacristán et al., 2025) [15]
	Primary producers	Terrestrial plant (dicot)	*Rumex crispus* (curled dock)	3-11	EC_{50}	186.79	(Sacristán et al., 2025) [15]
	Primary producers	Terrestrial plant (dicot)	*Rumex crispus* (curled dock)	3-11	EC_{10}	24.12	(Sacristán et al., 2025) [15]
	Primary producers	Terrestrial plant (dicot)	*Anagallis arvensis* (scarlet pimpernel)	3-11	EC_{50}	46.02	(Sacristán et al., 2025) [15]
	Primary producers	Terrestrial plant (dicot)	*Anagallis arvensis* (scarlet pimpernel)	3-11	EC_{10}	5.00	(Sacristán et al., 2025) [15]
	Primary consumers	Crustacean	*Ceriodaphnia silvestrii*	7	EC_{10}	0.04	(Freitas et al., 2018) [42]
	Primary consumers	Crustacean	*Ceriodaphnia silvestrii*	7	EC_{50}	0.30	(Freitas et al., 2018) [42]
	Primary consumers	Crustacean	*Ceriodaphnia dubia*	2	EC_{50}	18.65	(Isidori et al., 2005) [80]
	Primary consumers	Crustacean	*Ceriodaphnia dubia*	7	EC_{50}	0.18	(Isidori et al., 2005) [80]
	Primary consumers	Crustacean	*Daphnia magna*	2	LC_{50}	22.64	(Isidori et al., 2005) [80]
	Primary consumers	Crustacean	*Daphnia magna*	1	EC_{50}	831.6	(Park and Choi, 2008) [88]
	Primary consumers	Crustacean	*Daphnia magna*	2	EC_{50}	621.2	(Park and Choi, 2008) [88]
	Primary consumers	Crustacean	*Daphnia magna*	21	EC_{50}	46.2	(Wollenberger et al., 2000) [69]
	Primary consumers	Crustacean	*Daphnia magna*	21	EC_{10}	7.4	(Wollenberger et al., 2000) [69]
	Primary consumers	Crustacean	*Daphnia magna*	21	EC_{50}	86	(Zounková et al., 2011) [84]
	Primary consumers	Crustacean	*Daphnia magna*	2	EC_{50}	20	(Zounková et al., 2011) [84]
	Primary consumers	Crustacean	*Daphnia magna*	2	EC_{50}	114	(Kołodziesjka et al., 2013) [79]
	Primary consumers	Crustacean	*Daphnia magna*	2	EC_{10}	197	(Kolar et al., 2014) [57]

Cmpd.	Trophic level	Phylum/organism group	Species (common name)	Time (days)	Endpoint (effect assessed)	Effect (mg L^{-1})	Reference
	Primary consumers	Crustacean	*Daphnia magna*	2	EC_{50}	669	(Kolar et al., 2014) [57]
	Primary consumers	Rotifer	*Brachionus calyciflorus*	3	EC_{50}	1.87	(Isidori et al., 2005) [80]
	Primary consumers	Crustacean	*Thamnocephalus platyurus*	1	LC_{50}	25.00	(Isidori et al., 2005) [80]
	Primary consumers	Crustacean	*Artemia parthenogenetica*	1	LC_{50}	870.47	(Ferreira et al., 2007) [86]
	Primary consumers	Crustacean	*Artemia parthenogenetica*	2	LC_{50}	805.99	(Ferreira et al., 2007) [86]
	Primary consumers	Crustacean	*Moina macrocopa*	1	EC_{50}	137.1	(Park and Choi, 2008) [88]
OTC	Primary consumers	Crustacean	*Moina macrocopa*	2	EC_{50}	126.7	(Park and Choi, 2008) [88]
	Primary consumers	Crustacean	*Macrobrachium lanchesteri*	2	EC_{50}	300	(Rico et al., 2014) [89]
	Primary consumers	Annelida	*Limnodrilus hoffmeisteri*	2	EC_{50}	217	(Rico et al., 2014) [89]
	Primary consumers	Mollusca	*Melanoides tuberculata*	2	EC_{50}	958	(Rico et al., 2014) [89]
	Primary consumers	Mollusca	*Physella acuta*	2	EC_{50}	791	(Rico et al., 2014) [89]
	Primary consumers	Echinoderm	*Paracentrotus lividus*	2	EC_{10}	21.8	(Carballeira et al., 2012) [90]
	Primary consumers	Echinoderm	*Paracentrotus lividus*	2	EC_{50}	12	(Gharred et al., 2016) [91]
	Primary consumers	Echinoderm	*Arbacia lixula*	2	EC_{10}	85.1	(Carballeira et al., 2012) [90]
	Secondary consumers	Fish	*Labeo rohita*	15	NOEC	80	(Ambili et al., 2013) [92]
	Secondary consumers	Fish	*Oryzias latipes*	2	LC_{50}	215.4	(Park and Choi, 2008) [88]
	Secondary consumers	Fish	*Oryzias latipes*	4	LC_{50}	110.1	(Park and Choi, 2008) [88]
	Secondary consumers	Fish	*Danio rerio*	3	EC_{50}	127.6	(Oliveira et al., 2013) [93]
	Secondary consumers	Fish	*Clarias batrachus*	4	LC_{50}	663.8	(Pandit and Priya, 2020) [94]
	Secondary consumers	Fish	*Salvelinus namaycush*	1	LC_{50}	<200	(Marking et al., 1988) [71]
	Secondary consumers	Fish	*Piaractus mesopotamicus*	2	LC_{50}	7.6	(Carraschi et al., 2011) [95]
	Primary producers	Terrestrial plant (dicot)	*Raphanus sativus* L. (radish)	9-16	EC_{50}	40 mg kg^{-1}	(Parente et al., 2018) [96]
	Primary producers	Terrestrial plant (monoc)	*Lolium perenne* (ryegrass)	9-16	EC_{50}	113 mg kg^{-1}	(Parente et al., 2018) [96]
	Primary producers	Terrestrial plant (monoc)	*Allium cepa* (onion)	9-16	EC_{50}	112 mg kg^{-1}	(Parente et al., 2018) [96]
	Primary producers	Terrestrial plant (dicot)	*Daucus carota* (carrot)	6	EC_{50}	9.11 mg kg^{-1}	(Tasho et al., 2020) [97]
	Primary producers	Terrestrial plant (dicot)	*Daucus carota* (carrot)	15	EC_{50}	61.66 mg kg^{-1}	(Tasho et al., 2020) [97]
	Primary producers	Terrestrial plant (dicot)	*Capsicum annuum* (pepper)	6	EC_{50}	28.06 mg kg^{-1}	(Tasho et al., 2020) [97]
	Primary producers	Terrestrial plant (dicot)	*Capsicum annuum* (pepper)	15	EC_{50}	113.06 mg kg^{-1}	(Tasho et al., 2020) [97]
	Primary producers	Terrestrial plant (dicot)	*Lactuca sativa* (lettuce)	6	EC_{50}	13.15 mg kg^{-1}	(Tasho et al., 2020) [97]

Cmpd.	Trophic level	Phylum/organism group	Species (common name)	Time (days)	Endpoint (effect assessed)	Effect (mg L^{-1})	Reference
	Primary producers	Terrestrial plant (dicot)	*Lactuca sativa* (lettuce)	15	EC_{50}	97.83 mg kg^{-1}	(Tasho et al., 2020) [97]
	Primary consumers	Annelida	*Enchytraeus crypticus* (enchytraeid)	21	NOEC	2000 mg kg^{-1}	(Baguer et al., 2000) [43]
	Primary consumers	Annelida	*Enchytraeus crypticus* (enchytraeid)	21	EC_{10}	134 mg kg^{-1}	(Baguer et al., 2000) [43]
	Primary consumers	Annelida	*Aporrectodea caliginosa* (earthworm)	21	NOEC	3000 mg kg^{-1}	(Baguer et al., 2000) [43]
	Primary consumers	Annelida	*Aporrectodea caliginosa* (earthworm)	21	EC_{10}	1954 mg kg^{-1}	(Baguer et al., 2000) [43]
	Primary consumers	Annelida	*Aporrectodea caliginosa* cocoons	21	EC_{10}	2127 mg kg^{-1}	(Baguer et al., 2000) [43]
OTC	Primary consumers	Arthropoda	*Folsomia fimetaria* (springtail)	21	NOEC	≥5000 mg kg^{-1}	(Baguer et al., 2000) [43]
	Primary consumers	Annelida	*Eisenia fetida* tail (earthworm)	30	EC_{50}	51.661 mg kg^{-1}	(Baguer et al., 2000) [43]
	Primary consumers	Annelida	*Eisenia fetida* head (earthworm)	30	EC_{50}	20.387 mg kg^{-1}	(Baguer et al., 2000) [43]
EOTC	Non-trophic	Bacteria	Aerobic activated sludge bacterial community	2	EC_{50}	0.27	(Halling-Sørensen et al., 2002) [55]
	Primary producers	Green algae	-	4	EC_{50}	85.7	(Wu et al., 2024) [a] [7]
CTC	Primary producers	Cyanobacteria	*Microcystis aeruginosa*	4	EC_{50}	0.05	(Halling-Sørensen, 2000) [55]
	Primary producers	Cyanobacteria	*Microcystis aeruginosa*	4	EC_{10}	1.86	(Ye et al., 2017) [56]
	Primary producers	Cyanobacteria	*Microcystis aeruginosa*	4	EC_{20}	4.09	(Ye et al., 2017) [56]
	Primary producers	Cyanobacteria	*Microcystis aeruginosa*	10	NOEC	<0.500	(Guo and Chen, 2012) [98]
	Primary producers	Chlorophyta	*Selenastrum capricornutum*	7	EC_{50}	3.1	(Halling-Sørensen, 2000) [55]
	Primary producers	Chlorophyta	*Pseudokirchneriella subcapitata*	3	EC_{50}	1.8	(Yang et al., 2008) [60]
	Primary producers	Chlorophyta	*Pseudokirchneriella subcapitata*	3	NOEC	0.5	(Yang et al., 2008) [60]
	Primary producers	Chlorophyta	*Pseudokirchneriella subcapitata*	3	LOEC	1	(Yang et al., 2008) [60]
	Primary producers	Chlorophyta	*Pseudokirchneriella subcapitata*	4	EC_{50}	1.19	(Carusso et al., 2018) [82]
	Primary producers	Chlorophyta	*Pseudokirchneriella subcapitata*	3	EC_{50}	3.1	(Halling-Sørensen, 2000) [55]
	Primary producers	Chlorophyta	*Ankistrodesmus fusiformis*	4	EC_{50}	3.23	(Carusso et al., 2018) [82]
	Primary producers	Chlorophyta	*Chlorella pyrenoidosa*	4	EC_{50}	35.5	(Lu et al., 2015) [99]
	Primary producers	Cryptophyta	*Rhodomonas salina*	7	EC_{50}	0.11	(Holten Lützhøft et al., 1999) [75]
	Primary producers	Aquatic plant	*Lemna gibba*	7	EC_{10}	0.036	(Brain et al., 2004) [46]
	Primary producers	Aquatic plant	*Lemna gibba*	7	EC_{50}	0.65	(Brain et al., 2004) [46]
	Primary producers	Aquatic plant	*Lemna gibba*	1	EC_{50}	0.219	(Brain et al., 2004) [46]
	Primary producers	Terrestrial plant	*Daucus carota* (carrot)	7	EC_{50}	1.141	(Hillis et al., 2011) [39]

Cmpd.	Trophic level	Phylum/organism group	Species (common name)	Time (days)	Endpoint (effect assessed)	Effect (mg L^{-1})	Reference
	Primary producers	Terrestrial plant	*Daucus carota* (carrot)	7	EC_{10}	0.0031	(Hillis et al., 2011) [39]
	Primary producers	Terrestrial plant	*Lactuca sativa* (lettuce)	5	EC_{10}	0.0056	(Hillis et al., 2011) [39]
	Primary producers	Terrestrial plant	*Medicago sativa* (alfalfa)	5	EC_{10}	0.0072	(Hillis et al., 2011) [39]
	Primary producers	Terrestrial plant	*Arabidopsis thaliana*	28	EC_{10}	8.1	(Newborn et al., 2025) [100]
	Primary producers	Terrestrial plant	Rice	4-5	EC_{50}	39	(Liu et al., 2009) [40]
	Primary producers	Terrestrial plant	Rice	4-5	EC_{10}	8	(Liu et al., 2009) [40]
	Primary producers	Terrestrial plant	*Cucumis sativus* L. (cucumber)	4-5	EC_{50}	16	(Liu et al., 2009) [40]
	Primary producers	Terrestrial plant	*Cucumis sativus* L. (cucumber)	4-5	EC_{10}	0.2	(Liu et al., 2009) [40]
CTC	Primary producers	Terrestrial plant	*Cucumis sativus* L. (cucumber)	4-5	EC_{50}	48	(Liu et al., 2009) [40]
	Primary consumers	Crustacean	*Daphnia magna*	21	NOEC	1.23	(Ji et al., 2012) [101]
	Primary consumers	Crustacean	*Daphnia magna*	21	NOEC	0.41	(Ji et al., 2012) [101]
	Primary consumers	Crustacean	*Daphnia magna*	21	NOEC	16.7	(Ji et al., 2012) [101]
	Primary consumers	Crustacean	*Daphnia magna*	2	EC_{50}	225	(Park and Choi, 2008) [88]
	Primary consumers	Crustacean	*Daphnia magna*	2	EC_{50}	127.43	(Ji et al., 2012) [101]
	Primary consumers	Crustacean	*Moina macrocopa*	2	EC_{50}	153.04	(Ji et al., 2012) [101]
	Primary consumers	Crustacean	*Moina macrocopa*	2	EC_{50}	272	(Park and Choi, 2008) [88]
	Secondary consumers	Planarian	*Dugesia japonica*	1	LC_{50}	144.6	(Li, 2013) [70]
	Secondary consumers	Planarian	*Dugesia japonica*	2	LC_{50}	141.4	(Li, 2013) [70]
	Secondary consumers	Planarian	*Dugesia japonica*	3	LC_{50}	137.6	(Li, 2013) [70]
	Secondary consumers	Planarian	*Dugesia japonica*	4	LC_{50}	137.6	(Li, 2013) [70]
	Secondary consumers	Fish	*Oryzias latipes*	4	LC_{50}	78.9	(Park and Choi, 2008) [88]
	Primary producers	Terrestrial plant	*Cucumis sativus* L. (cucumber)	20	EC_{10}	300 mg kg^{-1}	(Liu et al., 2009) [40]
	Primary producers	Terrestrial plant	*Cucumis sativus* L. (cucumber)	20	EC_{50}	19 mg kg^{-1}	(Liu et al., 2009) [40]
	Primary consumers	Invertebrates	*Eisenia fetida* juveniles (earthworm)	28	EC_{50}	96.1 mg kg^{-1}	(Lin et al., 2012) [46]
	Primary consumers	Invertebrates	*Eisenia fetida* juveniles	28	NOEC	2.48 mg kg^{-1}	(Lin et al., 2012) [46]
	Primary consumers	Invertebrates	*Eisenia fetida* Cocoon	28	EC_{50}	120.3 mg kg^{-1}	(Lin et al., 2012) [46]
	Primary consumers	Invertebrates	*Eisenia fetida* Cocoon	28	NOEC	3.37 mg kg^{-1}	(Lin et al., 2012) [46]
ECTC	Non-trophic	Bacteria	Aerobic activated sludge bacterial community	2	EC_{50}	1.65	(Halling-Sørensen et al., 2002) [55]
	Primary producers	Green algae	-	4	EC_{50}	15.2	(Wu et al., 2024) [a] [7]

Cmpd.	Trophic level	Phylum/organism group	Species (common name)	Time (days)	Endpoint (effect assessed)	Effect (mg L^{-1})	Reference
ICTC	Non-trophic	Bacteria	Aerobic activated sludge bacterial community	2	EC_{50}	36.3	(Halling-Sørensen et al., 2002) [55]
	Primary producers	Green algae	-	4	EC_{50}	1.74	(Wu et al., 2024) [a] [7]
DOX	Primary producers	Green algae	*Pseudokirchneriella subcapitata*	2	EC_{50}	0.85	(Fu et al., 2017) [85]
	Primary producers	Green algae	*Pseudokirchneriella subcapitata*	3	EC_{50}	0.34	(Fu et al., 2017) [85]
	Primary producers	Green algae	*Tetraselmis chuii*	4	EC_{50}	14	(Prata et al., 2018) [102]
	Primary producers	Green algae	*Scenedesmus obliquus*	4	EC_{50}	16,214	(Liu et al., 2017) [103]
	Primary producers	Aquatic plant	*Lemna gibba*	7	EC_{50}	0.316	(Brain et al., 2004) [46]
	Primary producers	Aquatic plant	*Lemna gibba*	7	EC_{10}	0.054	(Brain et al., 2004) [46]
	Primary producers	Aquatic plant	*Lemna gibba*	7	NOEC	0.100	(Brain et al., 2004) [46]
DOX	Primary consumers	Annelida	*Eisenia fetida* (earthworm)	28	EC_{50}	96.1 mg kg^{-1}	(Lin et al., 2012) [46]
	Primary consumers	Annelida	*Eisenia fetida* (earthworm)	28	EC_{50}	120.3 mg kg^{-1}	(Lin et al., 2012) [46]
MNC	Primary producers	Cyanobacteria	*Microcystis aeruginosa*	12	EC_{20}	0.13	(Stoichev et al., 2011) [104]
	Primary producers	Cyanobacteria	*Microcystis aeruginosa*	12	EC_{50}	0.92	(Stoichev et al., 2011) [104]
	Secondary consumers	Fish	*Danio rerio*	3	LD_{50}	230	(F. Zhang et al., 2015) [105]
MTC	Primary producers	Green Algae	*Pseudokirchneriella subcapitata*	3	EC_{50}	127.4	(Zhang et al., 2022) [a] [106]

TPs are marked in italics. Yellow highlighting indicates the aquatic endpoint selected for EqP-derived $PNEC_{soil}$ calculation. Blue highlighting indicates chronic endpoints (EC_{10} or NOEC) from different aquatic trophic levels considered for the same compound during the selection process but not ultimately selected for $PNEC_{soil}$ derivation. Orange highlighting indicates the terrestrial endpoint selected for $PNEC_{soil}$ calculation. Green highlighting indicates chronic endpoints (EC_{10} or NOEC) from different terrestrial trophic level considered for the same compound during the selection process but not ultimately selected for $PNEC_{soil}$ calculation. [a]predicted endpoint. All toxicity values are expressed in mg L^{-1}, unless otherwise indicated. Cmpd.: compound.

Table S3. Physical-chemical properties of the target tetracyclines and transformation products.

**Compound (Abbreviation)**	**MW (g mol^{-1})**	**pK_a ^{a}**	**Log K_{OW} ^{a}**	**K_d ^{b} (L kg^{-1})**	**Molecular structure**
Tetracycline (TC)	444.44	3.30; 7.68; 9.69	-1.3	0.8818	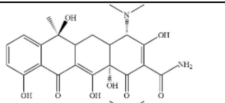
4-Epitetracycline (ETC)	444.15	4.8; 8.0	-1.33	0.8818	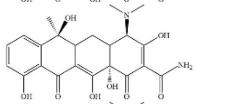
Anhydrotetracycline (ATC)	426.14	3.1; 7.5; 9.0	-	0.4732	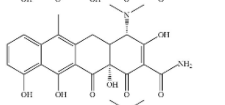
4-Epianhydrotetracycline (EATC)	426.14	-	-	0.4732	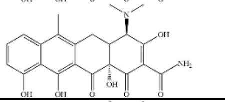
Oxytetracycline (OTC)	460.43	3.27; 7.3; 9.1	-0.90; -1.22	1.473	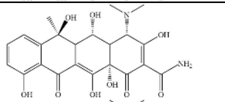
4-epioxytetracycline (EOTC)	460.15	4.71	-	1.473	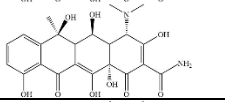
Chlortetracycline (CTC)	478.88	2.99; 7.4; 9.3	-0.62; -0.36; -2.9	1.4436	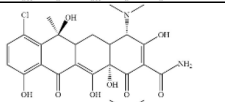
4-Epichlortetracycline (ECTC)	478.11	-	-	1.4436	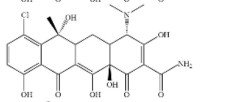
Isochlortetracycline (ICTC)	478.11	-	-	0.2	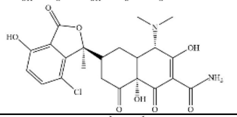
Methacycline (MTC)	478.11	3.5; 7.6; 9.2	-0.3	-	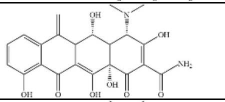
Doxycycline (DOX)	444.15	3.09; 7.7; 9.5	-0.54; 0.69	0.9872	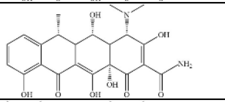
Minocycline (MNC)	457.18	2.8; 7.8; 9.5	0.05	-	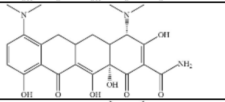
Demeclocycline* (DMC)	464.1	3.3; 7.2; 9.2	-1.14	-	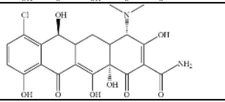

*Internal standard; TPs marked in italics. MW: molecular weight; pKa: acid dissociation constant; Kow: octanol-water partition coefficient. [a] (https://go.drugbank.com/); [b] (Wu et al., 2024 [7]).

Table S4. Recovery (R%). matrix effect (ME%), accuracy (A%) and precision of the method expressed as relative standard deviation (RSD) for composted sludge.

**Compound**	**Spike level**																**R^2**	**MQL (ng g^{-1} dw)**	**MDL (ng g^{-1} dw)**
	**5 ng g^{-1} dw**				**100 ng g^{-1} dw**				**500 ng g^{-1} dw**				**1000 ng g^{-1} dw**						
	R	ME	A	RSD	R	ME	A	RSD	R	ME	A	RSD	R	ME	A	RSD			
TC	100	-41	72	1.0	48	-31	83	3.8	69	-29	85	3.0	93	-15	95	3.2	0.995	3.00	0.9
ETC	107	-57	50	2.4	40	-49	86	3.8	48	-45	78	1.7	71	-30	104	3.5	0.997	10.5	3.2
ATC[1]	5	-68	122	11	17	-62	89	3.9	20	-60	106	3.1	19	-66	92	3.5	0.987	9.75	2.9
EATC[1]	20	-76	63	12	19	-78	84	5.6	21	-78	104	1.5	22	-83	88	1.5	0.986	6.31	1.9
OTC	95	-54	80	9.8	67	-39	108	4.3	58	-44	73	3.8	86	-37	101	3.6	0.997	4.84	1.5
EOTC	98	-56	66	0.9	35	-49	96	3.9	59	-50	70	4.2	80	-49	95	2.9	0.995	10.8	3.2
CTC	100	-37	61	2.6	95	-31	62	5.5	67	-29	68	2.8	98	-31	100	3.7	0.995	5.03	1.5
ECTC	100	-64	50	5.6	40	-69	66	7.6	56	-68	71	2.0	95	-74	89	7.8	0.994	7.99	2.4
ICTC[1]	142	-29	106	7.4	63	-33	70	3.9	87	-24	99	5.5	116	-42	98	1.8	0.995	1.22	0.37
MTC	102	-74	88	10	28	-49	94	3.2	39	-49	64	3.3	67	-50	93	3.3	0.995	3.39	1.0
DOX	98	-47	65	12	42	-37	104	2.6	53	-35	62	2.9	82	-37	95	2.8	0.996	4.13	1.2
MNC[1]	4	-75	43	3.5	26	-85	67	3.5	25	-79	75	9.7	72	-90	108	2.9	0.988	15.2	4.6

[1] Spike levels: 100, 500, 1000 and 2000 ng g^{-1} dw. TPs are written in italics.
